# New Fluorine-Containing Diamine Monomers for Potentially Improved Polyimides

**DOI:** 10.3390/molecules28196855

**Published:** 2023-09-28

**Authors:** Cassandra J. Hager, Colin D. McMillen, Rakesh Sachdeva, Arthur W. Martin, Joseph S. Thrasher

**Affiliations:** 1 Advanced Materials Research Laboratory, Department of Chemistry, Clemson University, 91 Technology Drive, Anderson, SC 29625, USA; cassandrajhager@gmail.com; 2 Hunter Laboratory, Department of Chemistry, Clemson University, 211 S. Palmetto Blvd., Clemson, SC 29634, USA; cmcmill@g.clemson.edu (C.D.M.); rakeshs@clemson.edu (R.S.); 3 R & D Technical Center, Daikin America, Inc., 2749 Hwy 20 West, Suite A, Decatur, AL 35601, USA; arthur.martin@dupont.com

**Keywords:** diamines, fluorine-containing monomers, polyimides, synthesis and characterization, crystallography, hydrogen bonding, two-step imidization

## Abstract

Two new fluorine-containing diamine monomers were designed with the goal of reducing charge transfer complex (CTC) interactions between neighboring chains in polyimides (i.e., high transparency/low color) while hopefully maintaining the well-known thermal stability and flexibility generally associated with polyimides. The proposed diamines have been prepared through (1) the functionalization of 1,3-bis[(pentafluorobenzyl)oxy]benzene with 4-aminophenol and (2) the addition of 2-chloro-5-nitrobenzotrifluoride to 4,4′-bicyclohexanol followed by reduction of the resulting dinitro compound. The new compounds have been characterized by multinuclear NMR and IR spectroscopy and high-resolution liquid chromatography-mass spectrometry as well as single-crystal X-ray diffraction on the new diamine prepared from 4,4′-bicyclohexanol. Not only was the structure of the proposed new diamine confirmed, but another interesting example of hydrogen bonding between an N-H proton and the π-system of an aromatic ring was observed and documented. Initial polymerizations have been carried out via the two-step imidization process.

## 1. Introduction

Flexible displays represent a current trend in the area of display technologies, and they often contain flexible organic light-emitting diodes (OLED). These displays are attractive due to their wide angle viewability, thin/lightweight features, and ability to bend. Unlike normal OLEDs, flexible OLEDs remove the rigid, glass substrate, replacing it with a flexible, polymeric material. Applications of flexible OLEDs can be seen in recent electronic releases, such as in modern flip phones and rollable smart televisions.

Flexible OLEDs require a polymer substrate that can withstand high-temperature processing and is optically clear. Other properties required of these materials include a high glass transition temperature (T_g_), optical transparency in the UV-Visible range (UV-Vis), solvent resistance, and low coefficient of thermal expansion (CTE). Commercially available polymer substrates that are optically transparent often fall short with T_g_s that are too low, e.g., poly(ethylene terephthalate), PET: 72 °C; poly(bisphenol A carbonate): 150 °C; and poly(ether-sulfone): 217 °C [1]. For the above application, polymer substrates should exhibit T_g_s above 300 °C. Currently, polyimides and polybenzazoles are a couple types of materials that can achieve such high T_g_s. Polybenzazoles have become less appealing for flexible OLEDs due to more limited availability of starting materials, along with several processing problems during manufacturing [2].

In contrast, polyimides (PI) are a widely used substrate in electronic technologies, due to their excellent thermal and mechanical properties as well as a straightforward two-step manufacturing process. Polyimides were first synthesized in 1908, through the use of 4-aminopthalic acid and were later commercialized in 1961 by DuPont, as the widely known Kapton^®^ [3,4,5]. Kapton^®^ is a high-molecular-weight polyimide synthesized from 4,4′-oxydianiline (ODA) and pyromellitic dianhydride (PMDA), which exhibits excellent thermal stability over a wide range of temperatures. This material opened the door for polyimides to be applied to various applications, including space technologies [6,7] and flexible electronic technologies [8,9].

Polyimides have also found wide applicability in the area of membrane-based gas separation, and fluorine-containing polyimides are finding an increasingly active role in this field [10]. Recently, Park and coworkers have reported that by reacting the surface of a non-fluorine-containing polyimide with a fluorine-containing diamine, they can increase both the permeability and selectivity of the membrane toward gas separation [10]. Meanwhile, Kim and coworkers fabricated a porous material by reacting a main-group metal porphyrin with a polyimide via sol–gel processing and further downstream steps, and they have demonstrated the potential applicability of the resulting porous material in environmental applications [11]. Thus, a wide range of potential applications await both the new fluorine-containing diamines that will be reported herein as well as any future polyimides therefrom.

The high thermal stability of polyimides originates as a result of the rigid imide–heterocyclic backbone. When imidization of poly(amic acid) to the polyimide film occurs, the possibility exists for a charge transfer complex (CTC) to form from the interaction of the electron-donating diamine with the electron-withdrawing dianhydride. Generally, CTC is seen in wholly aromatic polyimides. CTC interactions cause the polyimide backbones to be drawn tightly together, resulting in restricted movement of chains and higher T_g_s [12]. CTCs in polyimides cause strong absorption in the UV-Vis range, leading to a yellow-to-brown tinting and reduced transparency. In order to apply polyimides for flexible OLEDs, the CTCs need to be suppressed to produce colorless to nearly colorless polyimides (CPI).

Research efforts toward improving optical transparency through structural modifications of colorless PIs have mainly been accomplished through the design and synthesis of new monomers. The three main approaches include: (1) reduction of chain rigidity through the addition of alicyclic or aliphatic linkages [12,13,14], (2) decrease in the molecular packing and crystallinity of the polymer backbone by introducing side groups [12,14,15,16,17,18,19], and (3) copolymerization of more than two monomers within a polymerization [16]. The choice of approach is dictated by the various properties that may be affected by these adjustments. For example, rigid and highly conjugated substituents may be introduced into a material with the goal of increasing the thermal resistance; however, a potential trade-off may be the deterioration of the optical properties of the material.

In this study, two (2) new diamines are proposed that contain either perfluorinated aromatic rings or aromatic rings with perfluoroalkyl substituents, along with ether linkages and aliphatic/alicyclic chains. The hope is that the aliphatic/alicyclic chains and the ether linkages will disrupt the packing of the backbone, while maintaining thermal stability through the incorporation of fluorine-containing aromatic rings [15,17,20,21].

The reactivity of the new diamine monomers will be evaluated with commercial aromatic and aliphatic/alicyclic dianhydrides forming polyimides. These polyimides are hypothesized to exhibit optical transparencies and thermal stabilities similar to those seen in the literature [15,17,20,21]. This report will, however, focus on the design, preparation, and characterization of the new diamine monomers [22], while their polymerization into PIs will be detailed in a future publication.

## 2. Results and Discussion

### 2.1. Design of New Diamine Monomers (Monomers 1 and 2)

**Figure 1:**
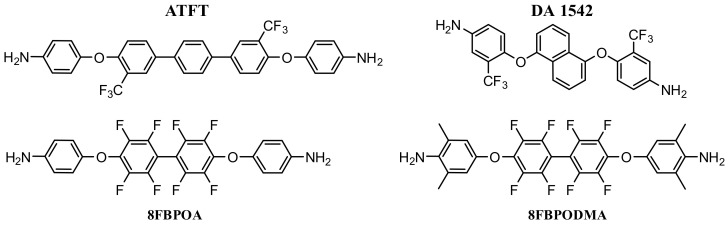
Diamine monomers with fluorine functionality (redrawn from concepts in references): ATFT [21], DA 1542 [15], 8FBPOA [18,20,25], and 8FBPODMA [18,20,25].

Previous work in our group in the field of fluorine chemistry directed our focus on the design of new diamine monomers containing fluorine, through the addition of either perfluorinated or -CF_3_ groups. PIs containing perfluorinated or -CF_3_ groups have been shown to reduce coloration of the resulting polymer while maintaining high thermal stability. The incorporation of these groups increases the free volume of PIs, allowing for the reduction of rigidity in the backbone [15,21,23]. Furthermore, PIs synthesized from diamines containing perfluorinated aromatic groups often exhibit good optical transmittance while maintaining high thermal stability with high T_g_s [18,19,24]. For the synthesis of novel fluorine-containing monomers, the properties of the monomers shown in Figure 1 were used as basis for molecular design and further comparison. 

**Figure 2:**
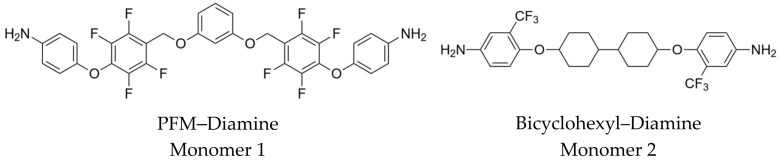
Two new monomers targeted for preparation herein.

The design of our first new diamine 4,4′-[1,3-bis[(2,3,5,6-tetrafluorobenzyl)oxy]benzene]bis(oxy)aniline (PFM–diamine, Monomer 1—see Figure 2) was based on extending a building block that one of us was familiar with from prior work, namely 1,3-bis[(pentafluorobenzyl)oxy]benzene (PFM) [26,27]. Mueller and coworkers used this compound or analogues/derivatives of it in polycondensation reactions to generate interesting polymeric materials [26,27,28,29], some of which were sulfonated to give polymer electrolytes for study as membranes in fuel cells, etc. [27]. Thus, the base monomer PFM was synthesized through a Williamson ether synthesis between the alkyl bromide of 2,3,4,5,6-pentafluorobenzyl bromide and the alkoxide of resorcinol. The alkoxide of resorcinol was generated via the use of potassium carbonate and a phase transfer catalyst, namely 18-crown-6. The generated alkoxide ion performs a backside attack on the alky halide, through an S_N_2 reaction mechanism, to provide the new ether linkage [26,28].

The PFM base monomer was functionalized with 4-aminophenol to form the PFM–diamine (Figure 2) in basic medium. Although the amino group is a stronger nucleophile in general, under alkaline conditions, the phenolate anion is formed, which preferentially removes a fluoride ion from the perfluorinated aromatic ring resulting in O- rather than N-arylation. The phenoxide ion is generated under a mildly alkaline medium using potassium carbonate in the presence of a phase transfer catalyst like 18-crown-6, which then attacks the perfluorinated aromatic ring at the position that produces the most stable Meisenheimer complex [30]. Oxygen-containing nucleophiles attacking perfluorinated aromatic rings have been shown to react predominantly at the position *para* to the benzyloxy group [28,31,32,33,34].

A second monomer, namely 4,4′-[[1,1′-bicyclohexyl]-4,4′-diylbis(oxy)]bis[2-(trifluoromethyl)aniline] (Bicyclohexyl–Diamine, Monomer 2—see Figure 2) was proposed through a collaboration with Daikin America. While the design of Monomer 1 focuses on providing flexibility and thermal stability, this diamine utilizes nonaromatic rings (specifically bicyclohexanedioxy groups), to help reduce the CTC interactions of the backbone of the resulting polyimide. The syntheses of structurally similar diamines incorporating 1,4-cyclohexanedioxy and 1,4-cyclohexanedimethaneoxy linkages have been reported in the literature [35,36]. The reported methods proceed via the reduction of the corresponding dinitro compounds that were synthesized from the reactions of 2-chloro-5-nitrobenzotrifluoride with the corresponding dialkoxides using sodium hydride (NaH) as base in *N,N*-dimethylformamide (DMF) [37]. The authors also reported organosoluble and transparent polyamides and polyimides of the cyclohexanol-based diamines [38,39]. During the course of our investigation, we also found two Chinese patents that describe the preparation of positional and structural isomers of our proposed diamine, namely the 3-amino-5-trifluoromethyl analogue [40] as opposed to the proposed 4-amino-2-trifluoromethyl isomer shown in Figure 2. In terms of a structural isomer, these workers also describe a diamine with 3,5-disubstituted cyclohexyl groups connected to a 4,4′-biphenolic core [41]. We have yet to see these results published in a scientific journal.

### 2.2. Preparation and Multinuclear NMR Spectroscopic Characterization of the New Diamine Monomers (Monomers 1 and 2)

1,3-Bis[(pentafluorobenzyl)oxy]benzene (PFM) was prepared by the literature method [28], and its synthesis was confirmed via multinuclear NMR and infrared spectra (see Figures S1–S4 in the Supporting Information—SI) as well as the melting point (SI). One notable difference is that only nine (9) resonances were visible in the ^13^C-NMR spectrum, rather than the twelve (12) originally reported, due to the large J-1 couplings to the three (3) carbon atoms with one fluorine atom each that split these resonances initially into large doublets, i.e., three doublets and six singlets equal 12 peaks. Both the percent yield (85%) and perhaps the purity were higher when compared to the prior literature reports [28], as evidenced by a slightly higher and sharper melting point.

**Scheme 1:**
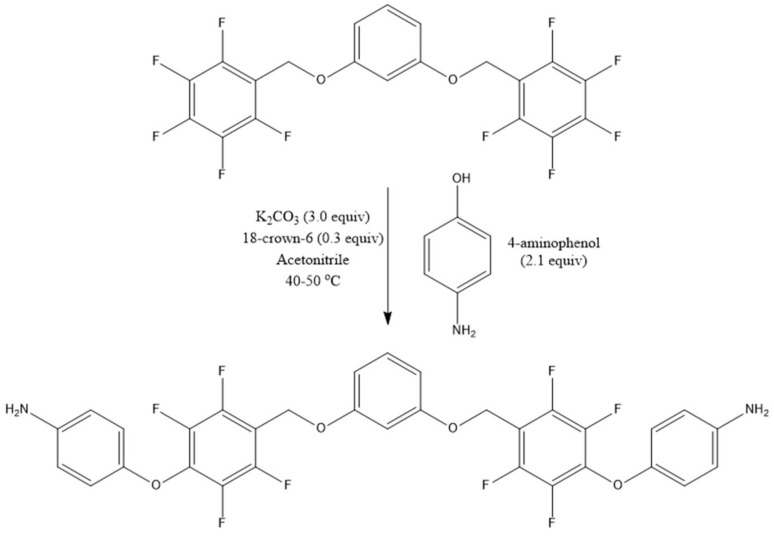
Functionalization of PFM with 4-aminophenol to give Monomer 1 (PFM–diamine).

The preparation of PFM–diamine was made following the reaction conditions shown in Scheme 1. For this reaction, acetonitrile was used as the solvent at a reaction temperature of 40–50 °C. The reaction proceeded for two days and was tracked by thin-layer chromatography (TLC) for completion. During the workup process, the mixture went through several color changes (from green to red) while being washed. After column chromatography, PFM–diamine was isolated as a pale tan solid. An initial attempt at preparing PFM–diamine under similar conditions using a solvent mixture of toluene and *N*,*N*-dimethylacetamide (DMAc) at 90–115 °C gave evidence for the desired product by TLC; however, the desired product could not be isolated.

**Figure 3:**
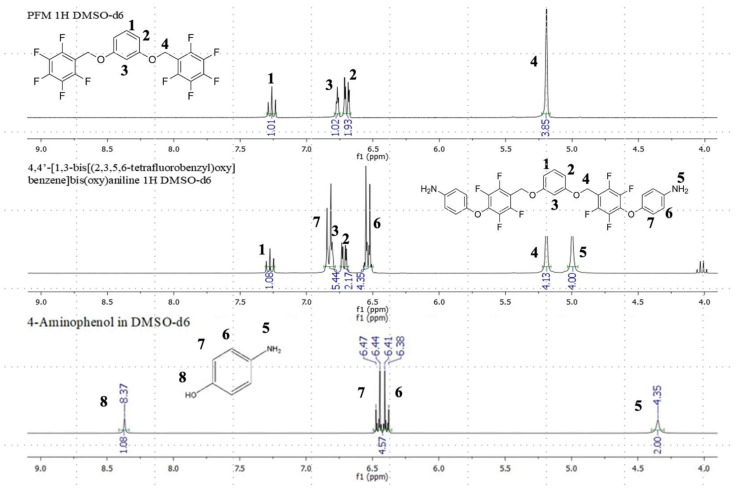
Comparison of ^1^H NMR spectra of PFM (**top**), PFM–diamine (**middle**), and 4-aminophenol (**bottom**).

**Figure 4:**
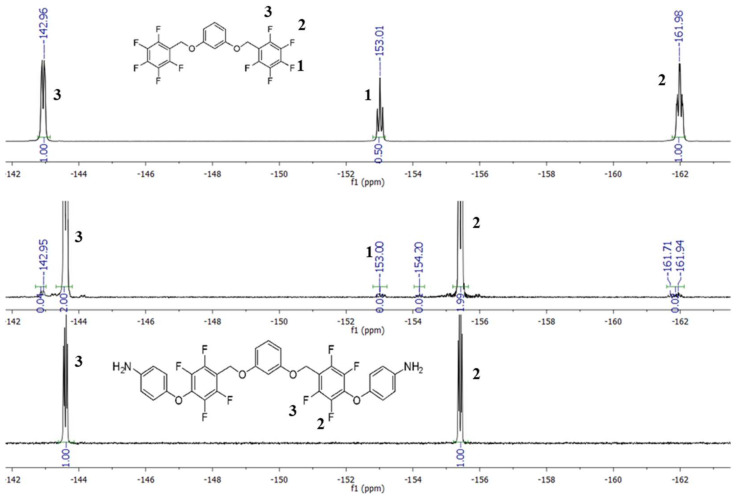
Comparison of ^19^F NMR spectra of PFM (**top**), impure PFM–diamine (**middle**), and pure PFM–diamine (**bottom**).

Characterization of the PFM–diamine by both ^1^H and ^19^F NMR spectroscopy clearly shows peaks consistent with the final product. The ^1^H NMR spectrum shows substitution of 4-aminophenol onto the fluorinated aromatic ring. The phenolic signal (8.37 ppm) completely disappears in the ^1^H NMR spectrum of the PFM–diamine, while the amino protons (5.00 ppm) remain. The resulting spectrum is consistent with expectation based upon a comparison of the proton spectra of the starting materials to that of the new PFM–diamine as shown in Figure 3. The ^19^F NMR spectrum confirms substitution of the aromatic ring at the unique *para*-fluorine position (see Figure 4). When comparing the ^19^F spectra of PFM, impure PFM–diamine, and purified PFM–diamine, the peak at −153 ppm due to the *para*-fluorine atom in PFM completely disappears. A shift in the signal of the *meta*-fluorine atoms due to the formation of the new ether linkage with 4-aminophenol is observed. The ^19^F spectrum of the impure sample of PFM–diamine clearly shows where the starting resonances of PFM are in comparison to the PFM–diamine. Peaks not associated with the starting material or product were also noticed and may be due to partially substituted PFM. The ^1^H, ^19^F, and ^13^C NMR spectra are available in the Supporting Information (Figures S5–S7).

**Scheme 2:**
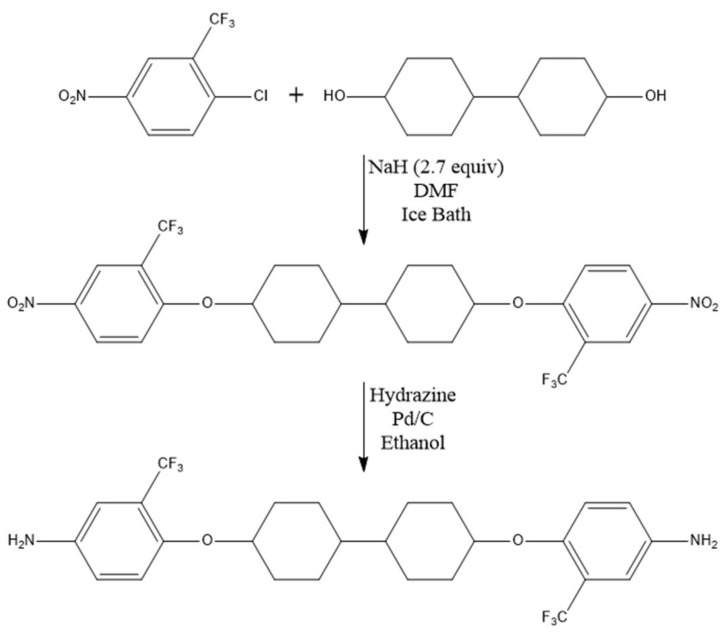
Preparation of the bicyclohexyl–dinitro compound, 1,1′-[[1,1′-bicyclohexyl]-4,4′-diylbis-(oxy)]bis[4-nitro-2-(trifluoromethyl)benzene] and bicyclohexyl–diamine, 4,4′-[[1,1′-bicyclohexyl]-4,4′-diylbis(oxy)]bis[2-(trifluoromethyl)aniline] (Monomer 2).

The bicyclohexyl-dinitro compound that is the precursor to Monomer 2 was prepared according to the reaction shown in Scheme 2, utilizing essentially the same procedure used to prepare the cyclohexyl-dinitro compounds mentioned above [35,36,37,38,39]. The dialkoxide of 1,4-bicyclohexanol was prepared using NaH as base in DMF at ice-bath temperature. The reaction mixture was allowed to warm towards room temperature, and 2-chloro-5-nitrobenzotrifluoride was added in portions using the ice bath to control the temperature of the reaction mixture. The reaction mixture changed to a brownish-colored solution, which could be stirred. After twenty hours, the resulting mixture was slowly poured into cold water and filtered. The precipitate was washed several times with cold ethanol and dried, yielding a yellow powder. The final material was recrystallized from hot ethanol, producing a light-yellow, powdery solid.

**Figure 5:**
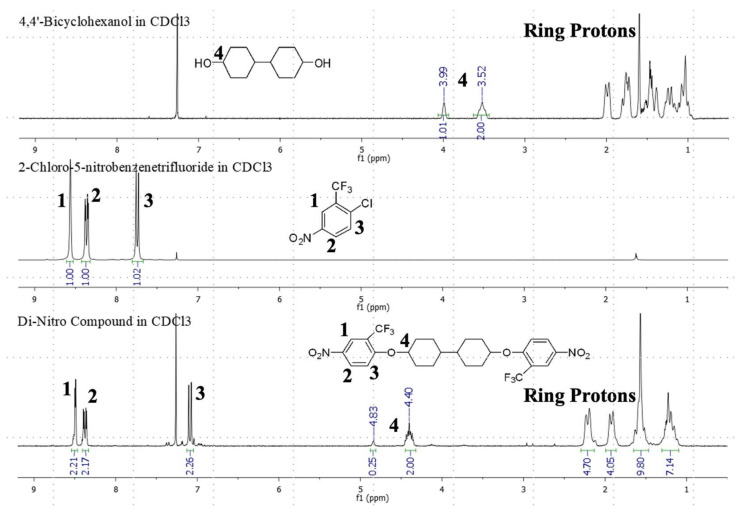
Comparison of ^1^H NMR spectra of starting materials to the bicyclohexyl–dinitro molecule, 1,1′-[[1,1′-bicyclohexyl]-4,4′-diylbis(oxy)]bis[4-nitro-2-(trifluoromethyl)benzene].

The bicyclohexyl–dinitro compound was analyzed by multinuclear NMR spectroscopy (see Figure 5 and Figures S10–S12). The ^1^H NMR spectrum of the product (Figure 5) versus those of the starting materials shows a shift of the aromatic H-3 proton as well as the disappearance of the alcoholic protons. The starting material 4,4′-bicyclohexanol is a mixture of *trans-trans*-, *cis-trans*-, and *cis-cis*-isomers, with the *trans-trans*-isomer being the dominant isomer [42,43,44]. Our proposed assignments of the *cis*- and *trans*-H-4 protons at 4.83 ppm and 4.40 ppm, respectively, suggest that the conversion of 4,4′-bicyclohexanol to the bicyclohexyl–dinitro compound and subsequent purification results in an even stronger preference for the *trans-trans*-isomer. On the other hand, the ^13^C NMR spectrum of the product (Figure S11) shows a number of sets of resonances that can only be rationalized by the presence of several isomers/ring conformations. Interestingly, in all of the aforementioned references on related chemistry involving either 1,4-cyclohexanol or 4,4′-bicyclohexanol, the authors never mention anything about isomers beyond pointing out the fact that their starting materials were a mixture of isomers. Nevertheless, when analyzing the ^19^F NMR spectrum (Figure S12), only a single resonance (singlet) is observed for the CF_3_ group at −62.33 ppm, as this functional group is sufficiently far enough from the cyclohexyl rings and remains unaffected by their conformations. 

**Figure 6:**
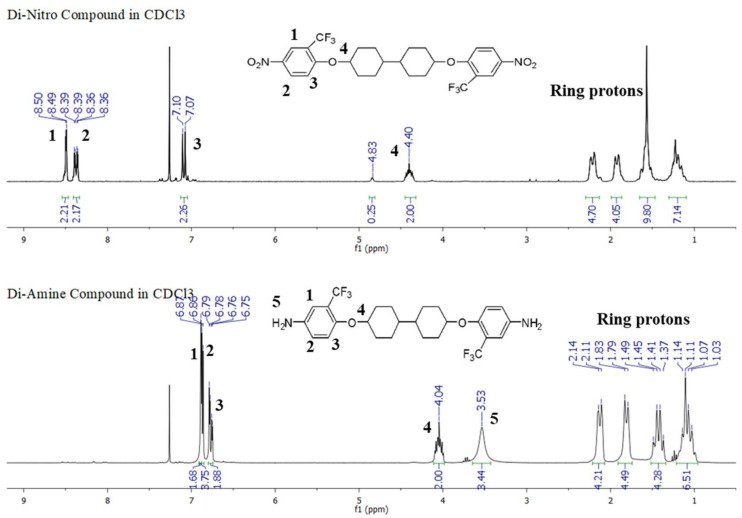
Comparison of ^1^H NMR spectra of the bicyclohexyl–dinitro molecule, 1,1′-[[1,1′-bicyclohexyl]-4,4′-diylbis(oxy)]bis[4-nitro-2-(trifluoromethyl)benzene], to the bicyclohexyl–diamine monomer, 4,4′-[[1,1′-bicyclohexyl]-4,4′-diylbis(oxy)]bis[2-(trifluoromethyl)aniline] after hydrogenation.

The hydrogenation of the bicyclohexyl–dinitro compound to the bicyclohexyl–diamine compound {Monomer 2, 4,4′-[[1,1′-bicyclohexyl]-4,4′-diylbis(oxy)]bis[2-(trifluoromethyl)aniline]} was easily accomplished utilizing Pd/C and hydrazine hydrate. This reaction was rather efficient and produced yellow crystals upon washing with cold ethanol and recrystallization from hexane. The ^1^H NMR spectrum of the bicyclohexyl–diamine is shown in comparison to that of the bicyclohexyl–dinitro compound below in Figure 6 as well as in Figure S14 along with the ^13^C and ^19^F NMR spectra in Figures S15 and S16, respectively. Upon reduction, the ^1^H NMR spectrum shows the appearance of the amino protons at 3.53 ppm, as well as an upfield shift of the resonances for the H-1 and H-2 aromatic protons due the electron donating effect of the amino functionality versus the electron-withdrawing effect of the nitro group on the aromatic ring. Recrystallization of the bicyclohexyl–diamine seemingly leads to a further preference of the *trans-trans*-isomer, especially when preparing single crystals suitable for X-ray diffraction studies (vide infra). Interestingly, Fan and coworkers recently prepared a series of polyethylene polymers containing 4,4′-bicyclohexanedioxy linkages via acyclic diene metathesis polymerization (ADMET), and from their DSC and X-ray diffraction studies, they concluded that only the *trans-trans*-isomeric groups are incorporated in the crystalline regions of their polymers, while the other isomers correspondingly are in the amorphous regions [45].

### 2.3. Further Characterization of the PFM–Diamine Monomer (Monomer 1), the Bicyclohexyl–Dinitro Compound, and the Bicyclohexyl–Diamine Monomer (Monomer 2), including the Crystal Structure of the Bicyclohexyl–Diamine Monomer (Monomer 2)

The ATR FT-IR spectra of all three new compounds reported herein as well as that of the known PFM monomer are consistent with the proposed structures and are shown in the Supporting Information (Figures S4, S8, S13 and S17).

With respect to high-resolution mass spectrometry (HRMS) liquid chromatographymass spectrometry (LC-MS), only one large signal was observed for each new diamine monomer by both the UV-Vis and MS detectors. Both diamines have two amino groups that can each pick up a proton and become charged. For the PFM–diamine (Monomer 1), the mass spectrum of the peak indeed shows the presence of both the singly charged [M + H]^+^ ion at *m*/*z* 649.1352 (theoretical *m*/*z* 649.1368) amu and the doubly charged [M + 2H]^2+^ ion at *m*/*z* 325.0720 (theoretical *m*/*z* 325.0731) amu (see Figure S9). Similarly, the mass spectrum of Monomer 2 (bicyclohexyl–diamine) shows both the singly charged [M + H]^+^ ion at *m*/*z* 517.2274 (theoretical *m*/*z* 517.2284) amu and the doubly charged [M + 2H]^2+^ ion at *m*/*z* 259.1178 (theoretical *m*/*z* 259.1178) amu (see Figure S18). The doubly charged ions were confirmed by the carbon-13 isotopic patterns that showed a spacing of 0.5 amu, indicating z = 2.

**Figure 7:**
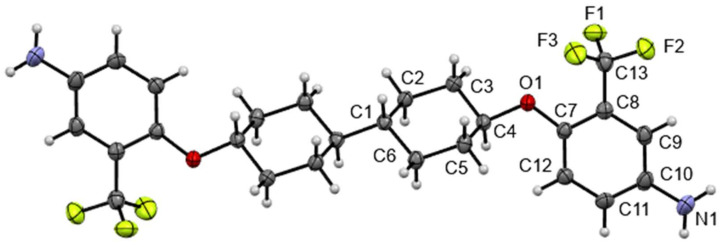
Molecular structure of the bicyclohexyl–diamine monomer 4,4′-[[1,1′-bicyclohexyl]-4,4′-diylbis(oxy)]bis[2-(trifluoromethyl)aniline], shown as 50% probability ellipsoids. Carbon atoms are gray, nitrogen atoms are blue, oxygen atoms are red, fluorine atoms are green, and hydrogen atoms are white.

The bicyclohexyl–diamine (C_26_H_30_F_6_N_2_O_2_, Monomer 2) was found to crystallize in the monoclinic space group *P*2_1/*c*_ with Z = 2, where half of the molecule is unique. The entire molecule is generated by inversion symmetry and is shown in Figure 7, where one can clearly see that we are dealing with the *trans*-*trans*-isomer. The cyclohexane rings are in a chair conformation forming the core of the molecule, with a dihedral angle (C2-C1-C1-C6) of 57.67(13)° joining the rings in plane with one another (though the individual cyclohexane rings are of course not planar). The chair conformation and dihedral angle are similar to other 4,4′-substituted bicyclohexane structures [45,46,47,48,49]. The end groups append to this central core through the ether bridge at a dihedral angle (H4-C4-O1-C7) of 45.13°, resulting in a mean-plane-to-mean-plane angle of 59.2(2)° between the end groups and the bicyclohexyl mean plane.

A search of the Cambridge Structural Database revealed 26 deposited structures having a 4,4′-substituted bicyclohexane fragment, but no comparable structures appear where that bicyclohexyl core is symmetrically flanked by bridging ethers. The structure of 4-(pent-3-enyl)-4′-ethoxy-1,1′-bicyclohexane (CSD refcode PAYYIX [50]) contains an ether bridge on one side of the molecule with comparable C-O bond lengths (1.425(5) Å and 1.393(6) Å versus 1.4473(18) Å and 1.3666(19) Å in C_26_H_30_F_6_N_2_O_2_) and a slightly more acute C-O-C angle (114.4(4)° versus 119.17(12)° in C_26_H_30_F_6_N_2_O_2_). The only other reported crystal structure of a bicyclohexyl core that is symmetrically 4,4′-substituted appears to be that of S,S′-(1,1′-bi(cyclohexyl)-4,4′-diylbis(methylene)) diethanethioate (CSD refcode LAHWEY, [51]). Molecules of the symmetric C_26_H_30_F_6_N_2_O_2_ monomer pack in two-dimensional sheets parallel to (1 0 2) via N-H···π interactions (N-H = 0.883(17) Å, H···centroid = 2.76(3) Å, N···centroid = 3.593(4) Å, N-H···centroid = 159(3)°; see SI, Figures S19 and S20). Specifically, the molecules are connected in an end-to-end fashion from the hydrogen bond donor NH_2_ group of one molecule to the π system of the (trifluoromethyl)aniline ring of a neighboring molecule. In this way, stacks of molecules are formed along the *b*-axis that extend into the (1 0 2) sheets due to the symmetric nature of the monomer. Both N-H···π and O-H···π aromatic hydrogen bonding interactions have been known for decades [52,53] and continue to be a topic of discussion [54].

### 2.4. Future Directions, including Initial Preparations of Poly(amic Acids) and Polyimides from Monomers 1 and 2

The preparation of polyimide films was attempted through the two-step thermal imidization method utilizing 4,4′-(hexafluoroisopropylidene)diphthalic anhydride (6FDA) as the dianhydride with each of the new diamines reported herein as well as with 2,2′-bis(trifluoromethyl)benzidine or 2,2′-bis(4-aminophenyl)hexafluoropropane in order to perfect our technique. The dianhydride 6FDA is a commonly utilized monomer for the synthesis of polyimides with new diamines [18,20,25]. The other two diamines were chosen due to their similar fluorine placement and aromatic nature as the newly synthesized diamines. Both NMP and DMAc were utilized as solvents in the preparation of poly(amic acid) solutions. Each polymerization was attempted in a dried sample vial equipped with a stir bar, rubber septum, and an argon balloon. Both the dianhydride and diamine monomers were dissolved separately and introduced into the reaction vial using a syringe. The solution exhibited a color change to yellow on the addition of the second monomer independent of which monomer was added. With stirring, the color dissipated, and the reaction solution became colorless. Reactions were further stirred for twenty-four hours in hopes of increasing the molecular weight of each polymer. Upon completion, the poly(amic acid) solutions were capped and stored in a refrigerator until thermal imidization could be carried out. Since this is an ongoing study, these reactions are currently being studied in order to ascertain the best imidization conditions [18,20,21,25]. Initially, we have been studying the imidization reactions via thermogravimetric analysis in order to track the conditions for the elimination of water. The results from this part of our study are planned for a future publication.

## 3. Materials and Methods

### 3.1. Materials

The following reagents were purchased from the listed vendors and used as received: 2,3,4,5,6-pentafluorobenzyl bromide and 18-crown-6 (SynQuest Laboratories, Alachua, FL, USA); 4-aminophenol, 4,4′-bicyclohexanol (mixture of isomers), 4,4′-(hexafluoroisopropylidene)-diphthalic anhydride (6FDA), 2,2′-bis(4-aminophenyl)hexafluoropropane, and 2,2′-bis(trifluoromethyl)benzidine (TCI America, Portland, OR, USA); acetone (Fisher Scientific, Atlanta, GA, USA); hydrazine hydrate (Alfa Aesar, Atlanta, GA, USA); 2-chloro-5-nitrobenzotrifluoride and sodium hydride (Sigma-Aldrich, St. Louis, MO, USA), LC-MS grade acetonitrile, water, and formic acid were procured from Fisher Scientific. Magnesium sulfate, potassium carbonate, potassium chloride, silica gel, resorcinol, acetonitrile, chloroform, deionized water, *N*,*N*-dimethylacetamide (DMAc), *N*,*N*-dimethylformamide (DMF), dimethyl sulfoxide, ethanol, ethyl acetate, hexane, *N*-methyl pyrrolidone (NMP), trichlorofluoromethane, and 5% Pd/C were taken from laboratory stock.

### 3.2. Instrumental Methods

Melting points were recorded on a Mel-Temp apparatus and left uncorrected. TLC was carried out on 0.25 mm Merck silica gel (60-F254).

^1^H- and ^19^F NMR spectra were recorded on a JEOL 300-MHz spectrometer at 301 and 283 MHz, respectively. ^13^C NMR spectra were recorded on a Bruker (Billerica, MA, USA) Avance 500 MHz NMR spectrometer at 125 MHz for carbon-13. CDCl_3_ or DMSO-*d_6_* was used as both a solvent and a reference for ^1^H- and ^13^C NMR spectroscopy, while CFCl_3_ was used as a reference for ^19^F NMR spectroscopy either internally or externally in a separate sample. The partial to complete assignments of the ^13^C NMR spectra were aided by short-range J-coupling to one or more fluorine atoms (in some cases), the presence or lack of a nuclear Overhauser effect (NOE), and additivity parameters for cyclohexyl and aromatic carbon atoms [55]. Infrared spectral measurements were recorded on a Nicolet (Atlanta, GA, USA) iS5 spectrometer with an iD5 ATR (attenuated total reflectance) accessory.

LC-MS analyses were performed on an Agilent (Santa Clara, CA, USA) 6545 Quadrupole Time-of-Flight LC-MS/MS system fitted with a dual AJS electrospray ionization (ESI) source. The QToF was operated in positive ion mode with the capillary voltage maintained at 3500 V, nebulizer pressure of 35 psi, evaporating gas maintained at 320 °C at a flow of 8 L/min, and the sheath gas maintained at 350 °C with a flow of 11 L/min. Mass spectra were recorded in profile mode using Agilent Masshunter 10.1 software. An Agilent 1290 II HPLC module interfaced to the mass spectrometer was used for separation and sample introduction into the source. The separation was performed in isocratic mode on an Agilent EclipsePlus C18 RRHD column (1.8 mm × 2.1 mm × 50 mm) maintained at 35 °C using a 70:30 mixture of acetonitrile/water with 0.1% formic acid modifier as the eluent system. An inline DAD detector set at 254 nm was used to detect the analyte prior to mass detection. The elution time on the MS is slightly delayed due to the extra length of tubing that connects the outlet of the DAD to the MS source.

Single-crystal X-ray diffraction data were measured at 100 K using a Bruker D8 Venture diffractometer equipped with Mo Kα radiation (Incoatec IμS, λ = 0.71073 Å) and a Photon 2 detector. The crystal was oscillated in 0.5° increments of phi and omega. Data were integrated and corrected for absorption (multi-scan) using the SAINT and SADABS algorithms in the Apex 3 (version 2017.3) software package [56]. The structure was solved by intrinsic phasing (SHELXT [57]) and subsequently refined by full-matrix least-squares techniques on *F*^2^ (SHELXL [58]). All non-hydrogen atoms were refined anisotropically. Hydrogen atoms attached to carbon atoms were placed in calculated positions using riding models with Ueq(H) = 1.2 Ueq(C). Hydrogen atoms of the NH_2_ group were identified from the difference electron density map and fully refined. Crystal Data for C_26_H_30_F_6_N_2_O_2_ (M = 516.52 g/mol): monoclinic, space group *P*2_1/*c*_ (no. 14), *a* = 13.0999(7) Å, *b* = 7.5533(4) Å, *c* = 12.8197(6) Å, *β* = 102.105(2)°, V = 1240.27(11) Å^3^, Z = 2, T = 100(2) K, μ(MoKα) = 0.118 mm^-1^, Dcalc = 1.383 g/cm^3^, 19,112 reflections measured (6.26° ≤ 2Θ ≤ 51.00°), 2290 unique (Rint = 0.0401, Rsigma = 0.0196), which were used in all calculations. The final R1 was 0.0390 (I > 2σ(I)) and wR2 was 0.0965 (all data). CCDC 2,288,210 contains the supplementary crystallographic data for this paper. These data can be obtained free of charge via https://www.ccdc.cam.ac.uk/structures/ (or from the CCDC, 12 Union Road, Cambridge CB2 1EZ, UK; Fax: +44-1223-336033; E-mail: deposit@ccdc.cam.ac.uk).

Thermogravimetric analyses (TGA) were conducted under a stream of dry nitrogen, using a TA Instruments (Newcastle, DE, USA) Model Q500.

### 3.3. Preparation of 4,4′-[1,3-bis[(2,3,5,6-tetrafluorobenzyl)oxy]benzene]bis(oxy)aniline (PFM–Diamine, Monomer 1)

A dry 250 mL three-necked, round-bottomed flask was equipped with a nitrogen inlet adapter, a bubbler, and a stir bar. PFM (2.00 g, 4.25 mmol), 4-aminophenol (1.39 g, 12.8 mmol, 3 equiv), potassium carbonate (1.76 g, 12.7 mmol, 3 equiv), 18-crown-6 (0.30 g, 1.15 mmol, 0.3 equiv), and acetonitrile (150 mL) were added under a stream of nitrogen. The mixture was heated at 50 °C for two days. The reaction was monitored by TLC for completion (60% hexane:40% ethyl acetate). Potassium carbonate was filtered out, and acetonitrile was removed under reduced pressure. The resulting brown oil was taken up in chloroform, producing a green solution with solids that were filtered out. The resulting filtrate was washed with deionized water (50 mL) until the aqueous layer was clear. The organic layer was dried with magnesium sulfate, and chloroform was removed under reduced pressure. The resulting solid was dissolved in a 60% hexane: 40% ethyl acetate solution and purified by silica gel column chromatography. The product was collected, and solvent removed under reduced pressure. The material was recrystallized from ethanol to produce light tan-colored crystals, mp 123.9–130.2 °C with decomposition (color change light brown powder to amber liquid). ^1^H NMR (301 MHz, DMSO-*d*_6_) δ 7.28 (t, 1H), 6.82 (t, *J* = 5.9 Hz, 1H), 6.72 (d, *J* = 8.2 Hz, 1H), 6.53 (d, *J* = 8.8 Hz, 1H), 5.19 (s, 1H), 5.00 (s, 1H). ^13^C NMR (125 MHz, DMSO-*d*_6_) δ 159.56 (s), 148.37 (s), 145.95 (m, ^1^*J*_C-F_ = 245.0 Hz), 145.89 (s), 141.42 (dd, ^1^*J*_C-F_ = 246.3 Hz), 135.31 (s), 130.82 (s), 117.05(s), 115.02 (s), 110.60 (t, ^2^*J*_C-F_ = 18.0 Hz), 108.42 (s), 102.27 (s), 58.03 (s). ^19^F NMR (283 MHz, DMSO-*d*_6_) δ −143.60 (dd, *J* = 23.1, 9.2 Hz), −155.41 (dd, *J* = 22.9, 9.2 Hz). FT-IR (ATR): $\tilde{\upsilon }$/cm^−1^ = 3637, 3397, 2956, 2921, 1589, 1494, and other frequencies (see Figure S8). HRMS (LC-MS, positive ion mode): *m*/*z* calcd for [C_32_H_20_F_8_N_2_O_4_ + H]^+^ 649.1368, found 649.1352; *m*/*z* calcd for [C_32_H_20_F_8_N_2_O_4_ + 2H]^2+^ 325.0731, found 325.0720.

### 3.4. Preparation of 1,1′-[[1,1′-bicyclohexyl]-4,4′-diylbis(oxy)]bis[4-nitro-2-(trifluoromethyl)benzene]

A dry 500 mL, three-necked, round-bottomed flask was equipped with a nitrogen inlet adapter, a condenser (topped with a bubbler), an addition funnel, and a stir bar. Sodium hydride (10.0 g, 417 mmol, 2.7 equiv) was then added under a stream of nitrogen. An ice bath was placed underneath the flask in order to keep the temperature of the reaction mixture below room temperature. *N*,*N*-Dimethylformamide (DMF, 270 mL) and 4,4′-bicyclohexanol (27.5 g., 139 mmol) were added directly to the flask. The mixture was stirred at 0 °C for two hours, and a gray-colored sludge formed. Dimethyl sulfoxide (DMSO, 80 mL) was added to the mixture, and the temperature of the reaction was allowed to warm to room temperature. *This is hazardous, as both DMF and DMSO can form explosive compounds with sodium hydride* [37]. As a precaution, the reaction mixture was cooled in an ice bath, and 2-chloro-5-nitrobenzotrifluoride (65.8 g, 43.1 mL, 292 mmol, 2.1 equiv) was added dropwise to the mixture using an addition funnel. Once this reagent was completely added, the solution was stirred under nitrogen for an additional twenty hours at 5 °C. The solution was then slowly added onto ice water. The solid was filtered and washed with cold ethanol several times. The final material was recrystallized from hot ethanol, leaving behind a light-yellow, powdery solid, mp 165.0–167.9 °C with decomposition (color change brown to dark reddish to amber at ca. 135 °C). ^1^H NMR (301 MHz, CDCl_3_) δ 8.50 (dd, *J* = 6.3, 2.7 Hz, 1H), 8.44–8.30 (m, 1H), 7.07 (t, *J* = 9.5 Hz, 1H), 4.83 (s, 1H), 4.40 (s, 1H), 2.18 (t, *J* = 16.0 Hz, 2H), 1.92 (d, *J* = 11.9 Hz, 2H), 1.70–1.44 (m, 4H), 1.36–1.10 (m, 3H). ^13^C NMR (125 MHz, DMSO-*d*_6_) δ 122.5 (q, ^1^*J*_C-F_ = 272 Hz) and other resonances. ^19^F NMR (283 MHz, CDCl_3_) δ −63.22 (s). FT-IR (ATR): $\tilde{\upsilon }$/cm^−1^ = 3124, 3091, 2946, 2865, 1617, 1519, 1488, 1334, 1280, 1116, 1047, 1014, 690, and other frequencies (see Figure S13).

### 3.5. Preparation of 4,4′-[[1,1′-bicyclohexyl]-4,4′-diylbis(oxy)]bis[2-(trifluoromethyl)aniline] (Bicyclohexyl–Diamine, Monomer 2)

A dry 150 mL three-necked, round-bottomed flask was equipped with a nitrogen inlet adapter, condenser, addition funnel, and a stir bar. The compound 1,1′-[[1,1′-bicyclohexyl]-4,4′-diylbis(oxy)]bis[4-nitro-2-(trifluoromethyl)benzene] (5.0 g, 8.6 mmol), 5% Pd/C (0.205 g), and ethanol (100 mL) were then added. Hydrazine hydrate (5 mL) was added via the addition funnel over a thirty-minute period at 80 °C. Once this addition was completed, the reaction was refluxed for three hours. After refluxing, the hot solution was immediately filtered to remove the Pd/C. The filtrate was concentrated, and the solids were filtered, recovered, and dried. The light-yellow crystals obtained were washed with cold ethanol and recrystallized from hexane for X-ray crystallography, mp 188.0–190.2 °C with slight decomposition (color change yellow to light orange at 185 °C). ^1^H NMR (301 MHz, CDCl_3_) δ 6.88 (s, 1H), 6.87 (d, *J* = 4.7 Hz, 1H), 6.77 (dd, *J* = 8.7, 2.8 Hz, 1H), 4.04 (s, 1H), 3.53 (s, 2H), 2.12 (d, *J* = 10.1 Hz, 2H), 1.81 (d, *J* = 10.7 Hz, 2H), 1.43 (dd, *J* = 23.7, 12.2 Hz, 2H), 1.09 (dd, *J* = 22.4, 11.8 Hz, 3H). ^13^C NMR (125 MHz, DMSO-*d*_6_) δ 145.89 (s), 142.47 (s), 124.01 (q, ^1^*J*_C-F_ = 272.5 Hz), 119.01 (q, ^2^*J*_C-F_ = 228.8 Hz), 118.59 (s), 117.71 (s), 111.31 (q, ^3^*J*_C-F_ = 2.5 Hz), 77.61 (s), 41.13 (s), 31.88 (s), 27.46 (s). ^19^F NMR (283 MHz, CDCl_3_) δ -63.22 (s). FT-IR (ATR): $\tilde{\upsilon }$/cm^−1^ = 3444, 3367, 2944, 2861, 1616, 1496, 1236, 1124, and other frequencies (see Figure S17). HRMS (LC-MS, positive ion mode): *m*/*z* calcd for [C_26_H_30_F_6_N_2_O_2_ + H]^+^ 517.2284, found 517.2274; [C_26_H_30_F_6_N_2_O_2_ + 2H]^2+^ 259.1178, found 259.1178.

## 4. Conclusions

The successful preparation of two novel diamines has been discussed. The PFM–diamine was prepared through the functionalization of PFM with 4-aminophenol, and its structure was confirmed by multinuclear NMR and IR spectroscopy and mass spectrometry. To the best of our knowledge, this represents the first diamine monomer prepared starting from PFM, and this new diamine monomer should find application in new polyimides, polyamides, and the like. A second diamine was prepared via the addition of 2-chloro-5-nitrobenzotrifluoride to 1,4-bicyclohexanol followed by reduction of the nitro groups to the diamine. The preparation of the bicyclohexyl–diamine was confirmed by multinuclear NMR and IR spectroscopy, mass spectrometry, and X-ray crystallography. The latter gives another example of hydrogen bonding between an N-H proton and the π-system of an aromatic ring. Both of these monomers would provide flexibility within a polyimide, while the bicyclohexyl–diamine would provide a nonaromatic system to reduce the CTC interactions. The preparation of new polyimides from these diamines versus benchmark polyimides from known fluorine-containing diamines has been initiated utilizing the two-step imidization process.

## Data Availability

All available data are presented either within this article or the supporting information in the Supplementary Materials.

## Supplementary Materials

The following supporting information can be downloaded at https://www.mdpi.com/article/10.3390/molecules28196855/s1. Synthesis of 1,3-Bis[(pentafluorobenzyl)oxy]benzene Monomer (PFM), Figures S1–S20, Tables S1–S3, References, and CIF and CheckCIF Files. References [28,55] are cited in the Supplementary Materials.

## Sample Availability

Samples of the compounds described above are not available from the authors.
