# Supplementary material for: New Fluorine-Containing Diamine Monomers for Potentially Improved Polyimides
Source: Molecules. 2023 Sep 28;28(19):6855. doi: 10.3390/molecules28196855 (PMC10574420; doi:10.3390/molecules28196855)
Supplement: Supplementary file 1 [file molecules-28-06855-s001.zip › molecules-2623743-supplementary.pdf]

New Fluorine-Containing Diamine Monomers for Potentially Improved Polyimides

Cassandra J. Hager [1,2], Colin D. McMillen [2], Rakesh Sachdeva [2], Arthur W. Martin [3], and Joseph S. Thrasher [1,2]*

[1] Department of Chemistry, Clemson University, Advanced Materials Research Laboratory, 91 Technology Drive, Anderson, South Carolina 29625 (USA); thrash5@clemson.edu
[2] Department of Chemistry, Clemson University, Hunter Laboratory, 211 S. Palmetto Blvd., Clemson, South Carolina 29634 (USA)
[3] Daikin America, Inc., R & D Technical Center, 2749 Hwy 20 West, Suite A, Decatur, Alabama 35601 (USA)
* Correspondence: thrash5@clemson.edu; Tel.: 01-205-310-3697

Table of Contents **Page**

1. Synthesis of 1,3-Bis[(pentafluorobenzyl)oxy]benzene Monomer (PFM) 3

2. Figures

Figure S1. ^{1}H NMR spectrum of 1,3-bis[(pentafluorobenzyl)oxy]benzene. 4
Figure S2. ^{13}C NMR spectrum of 1,3-bis[(pentafluorobenzyl)oxy]benzene. 5
Figure S3. ^{19}F NMR spectrum of 1,3-bis[(pentafluorobenzyl)oxy]benzene. 6
Figure S4. ATR FT-IR Spectrum of 1,3-bis[(pentafluorobenzyl)oxy]benzene. 7
Figure S5. ^{1}H NMR Spectrum of 4,4′-[1,3-bis[(2,3,5,6-tetrafluorobenzyl)oxy]benzene]bis(oxy)-aniline. 8
Figure S6. ^{13}C NMR spectrum of 4,4′-[1,3-bis[(2,3,5,6-tetrafluorobenzyl)oxy]benzene]bis(oxy)-aniline. 9
Figure S7. ^{19}F NMR Spectrum of 4,4′-[1,3-bis[(2,3,5,6-tetrafluorobenzyl)oxy]benzene]bis(oxy)-aniline. 10
Figure S8. ATR FT-IR Spectrum of 4,4′-[1,3-bis[(2,3,5,6-tetrafluorobenzyl)oxy]benzene]bis(oxy)-aniline. 11
Figure S9. HRMS (LC-MS) of 4,4′-[1,3-bis[(2,3,5,6-tetrafluorobenzyl)oxy]benzene]bis(oxy)-aniline. 12
Figure S10. ^{1}H NMR Spectrum of 1,1'-[[1,1'-bicyclohexyl]-4,4'-diylbis(oxy)]bis[4-nitro-2-(trifluoromethyl)benzene]. 13
Figure S11. ^{13}C NMR spectrum of 1,1'-[[1,1'-bicyclohexyl]-4,4'-diylbis(oxy)]bis[4-nitro-2-(trifluoromethyl)benzene]. 14
Figure S12. ^{19}F NMR Spectrum of 1,1'-[[1,1'-bicyclohexyl]-4,4'-diylbis(oxy)]bis[4-nitro-2-(trifluoromethyl)benzene]. 15
Figure S13. ATR FT-IR Spectrum of 1,1'-[[1,1'-bicyclohexyl]-4,4'-diylbis(oxy)]bis[4-nitro-2-(trifluoromethyl)benzene]. 16
Figure S14. ^{1}H NMR Spectrum of 4,4'-[[1,1'-bicyclohexyl]-4,4'-diylbis(oxy)]bis[2-(trifluoromethyl)-aniline] . 17
Figure S15. ^{13}C NMR spectrum of 4,4'-[[1,1'-bicyclohexyl]-4,4'-diylbis(oxy)]bis[2-(trifluoromethyl)-aniline]. 18
Figure S16. ^{19}F NMR Spectrum of 4,4'-[[1,1'-bicyclohexyl]-4,4'-diylbis(oxy)]bis[2-(trifluoromethyl)-aniline]. 19

Figure S17. ATR FT-IR Spectrum of 4,4'-[[1,1'-bicyclohexyl]-4,4'-diylbis(oxy)]bis[2-(trifluoromethyl)-aniline]. 20
Figure S18. HRMS (LC-MS) of 4,4'-[[1,1'-bicyclohexyl]-4,4'-diylbis(oxy)]bis[2-(trifluoromethyl)-aniline]. 21
Figure S19. Hydrogen Bonding in 4,4'-[[1,1'-bicyclohexyl]-4,4'-diylbis(oxy)]bis[2-(trifluoromethyl)-aniline]. 22
Figure S20. Unit cell packing diagram for 4,4'-[[1,1'-bicyclohexyl]-4,4'-diylbis(oxy)]bis[2-(trifluoro-methyl)aniline]. ($C_{26}H_{30}F_6N_2O_2$, monomer 2) viewed along the *b*-axis. 23

3. Tables

Table S1. Assignments of ^{13}C NMR spectrum of 1,3-bis[(pentafluorobenzyl)oxy]benzene from J-coupling to fluorine, NOEs, and additivity parameters. 24
Table S2. Assignments of ^{13}C NMR spectrum of 4,4′-[1,3-bis[(2,3,5,6-tetrafluorobenzyl) oxy]benzene]bis(oxy)aniline from J-coupling to fluorine, NOEs, and additivity parameters. 25
Table S3. Assignments of ^{13}C NMR spectrum of 4,4'-[[1,1'-bicyclohexyl]-4,4'-diyl bis(oxy)]bis[2-(trifluoromethyl)aniline] from J-coupling to fluorine, NOEs, and additivity parameters. 26

4. References 27

1. Synthesis of 1,3-Bis[(pentafluorobenzyl)oxy]benzene Monomer (PFM) [1].

This synthesis was performed as reported in Reference 1 [1]. A dry 1000-mL, three-necked, round-bottomed flask was equipped with a nitrogen inlet adapter, a bubbler, and a stir bar. Resorcinol (15.00 g, 136.2 mmol), 18-crown-6 (3.60 g, 13.6 mmol, 0.1 eq.), 2,3,4,5,6-pentafluorobenzyl bromide (43.21 mL, 286.1 mmol, 2.1 eq), and anhydrous acetone (680 mL) were added under a stream of nitrogen. After several minutes of mixing, K_2CO_3 (39.54 g, 286.1 mmol, 2.1 eq) was added to the mixture, which was stirred under nitrogen for 3-5 days at room temperature, while being checked by TLC (60% hexane:40% ethyl acetate). Once a clean TLC was produced, potassium carbonate was filtered from the solution via gravity filtration. The material was concentrated under reduced pressure until a solid was produced. The residual solid was taken up in dichloromethane and washed twice with deionized water and twice with 1 M aqueous potassium chloride solution. The organic layer was collected and dried with magnesium sulfate. The drying agent was then removed by filtration, and the resulting solution was concentrated under reduced pressure until solid formed. The solid was recrystallized in 90% hexanes/ 10% dichloromethane to give a pure product (54.4 g) in 85% yield; mp 86.8-87.5. ℃ (cf. mp 82-85 ℃ [1]). ^{1}H NMR (301 MHz, DMSO-d_6) δ 7.26 (t, J = 8.2 Hz, 1H), 6.77 (t, J = 2.2 Hz, 1H), 6.70 (dd, J = 8.2 Hz, 2H), 5.19 (s, 4H). ^{13}C NMR (125 MHz, DMSO-d_6) δ 159.40 (s), 145.59 (dt, $^{1}J_{C\text{-}F}$ = 248.2 Hz), 141.49 (m, $^{1}J_{C\text{-}F}$ = 246.3 Hz), 137.51 ($^{1}J_{C\text{-}F}$ = 247.5 Hz), 130.73 (s), 110.65 (td), 108.40 (s), 102.24 (s), 57.82 (s). ^{19}F NMR (283 MHz, DMSO-d_6) δ -142.93 (dd, J = 23.4 Hz), -153.03 (dd, J = 22.2 Hz), -162.00 (td, J = 23.1 Hz). FT-IR (ATR): $\tilde{\nu}$/cm^{-1} = 2958, 2892, 1590, 1504, 1151, 935, and other frequencies (see **Figure S4** and reference 1 [1]).

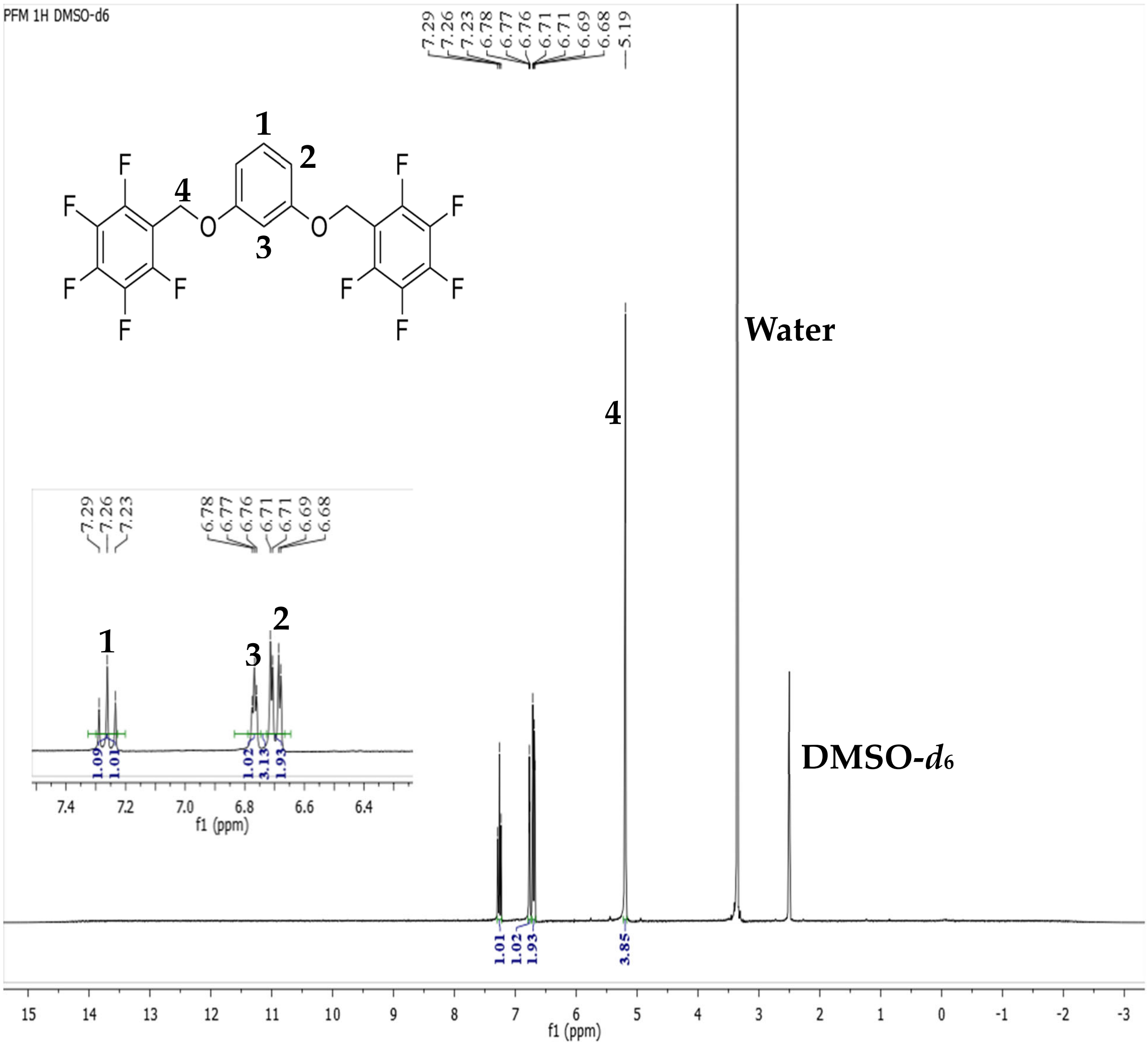

Figure S1. ^1H NMR spectrum of 1,3-bis[(pentafluorobenzyl)oxy]benzene.

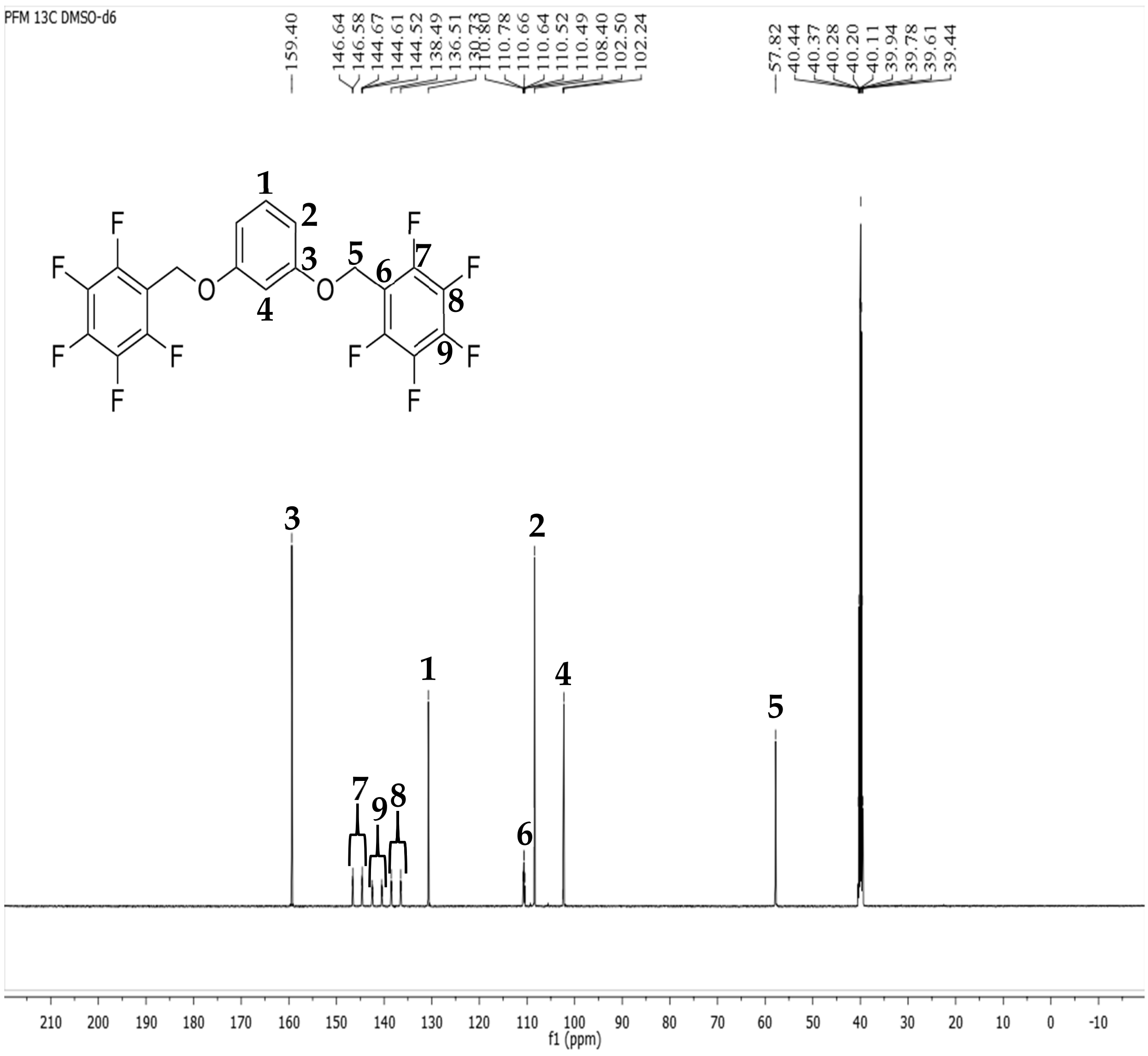

Figure S2. ^{13}C NMR spectrum of 1,3-bis[(pentafluorobenzyl)oxy]benzene.

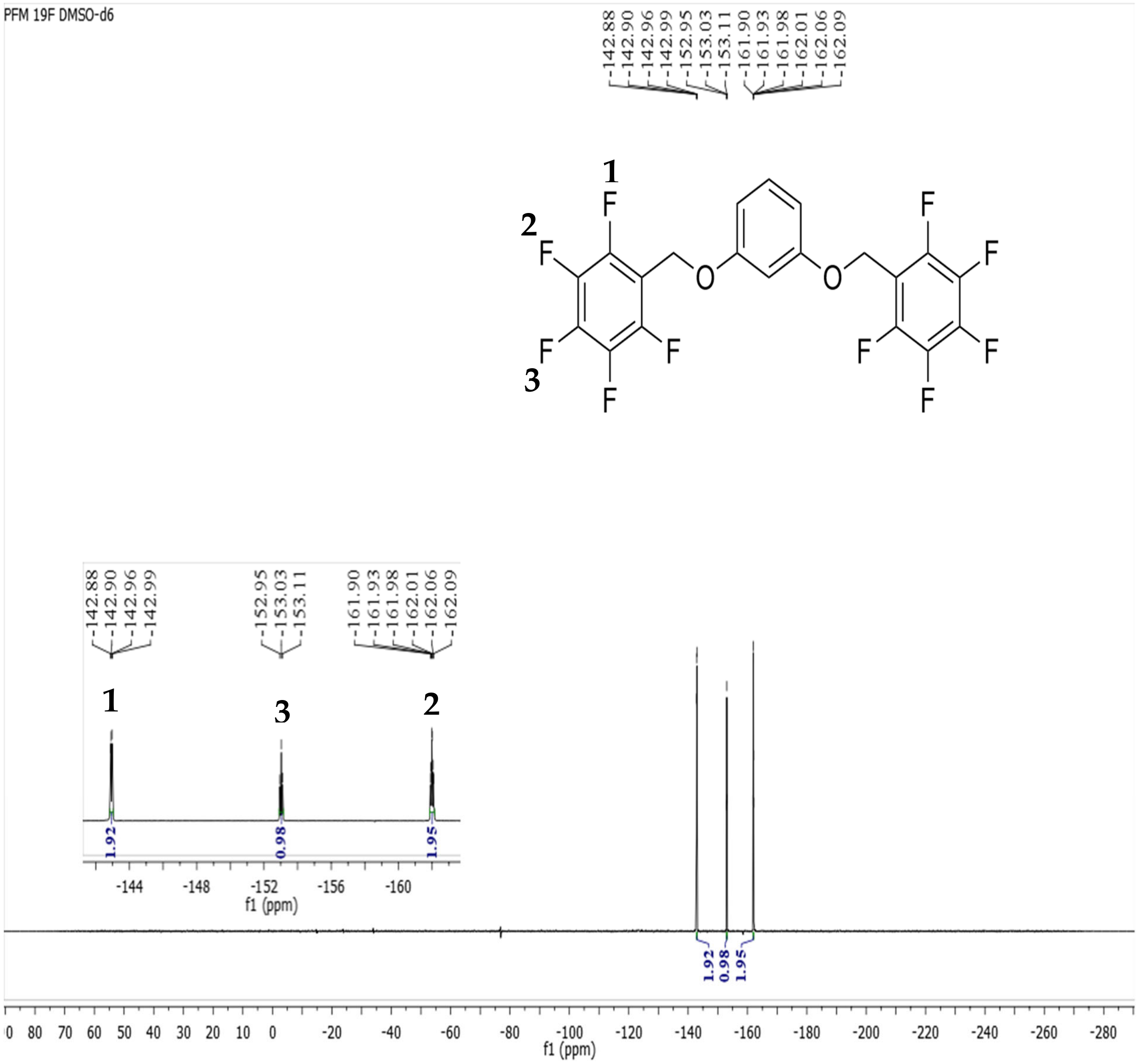

Figure S3. ^{19}F NMR spectrum of 1,3-bis[(pentafluorobenzyl)oxy]benzene.

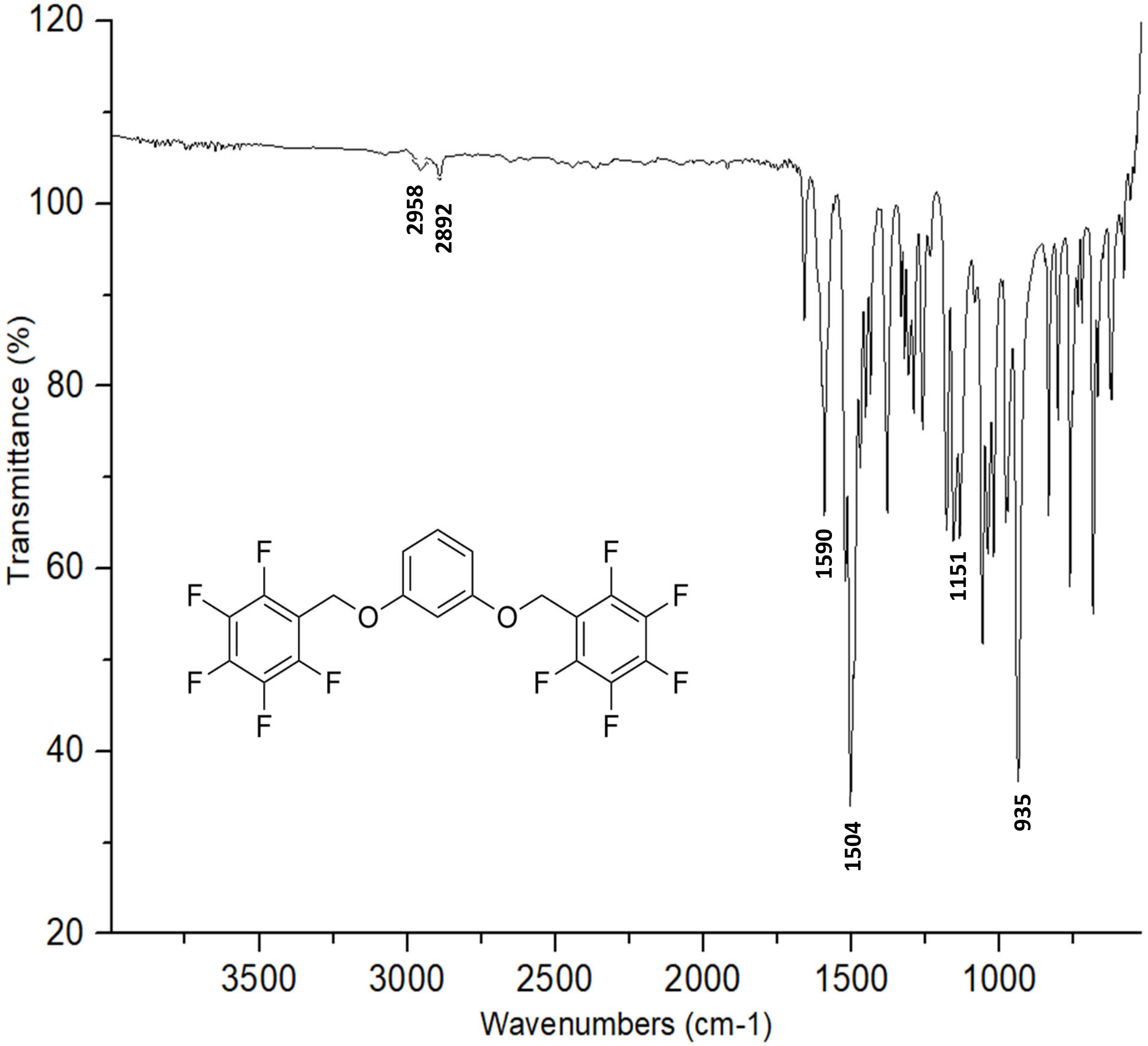

Figure S4. ATR FT-IR Spectrum of 1,3-bis[(pentafluorobenzyl)oxy]benzene.

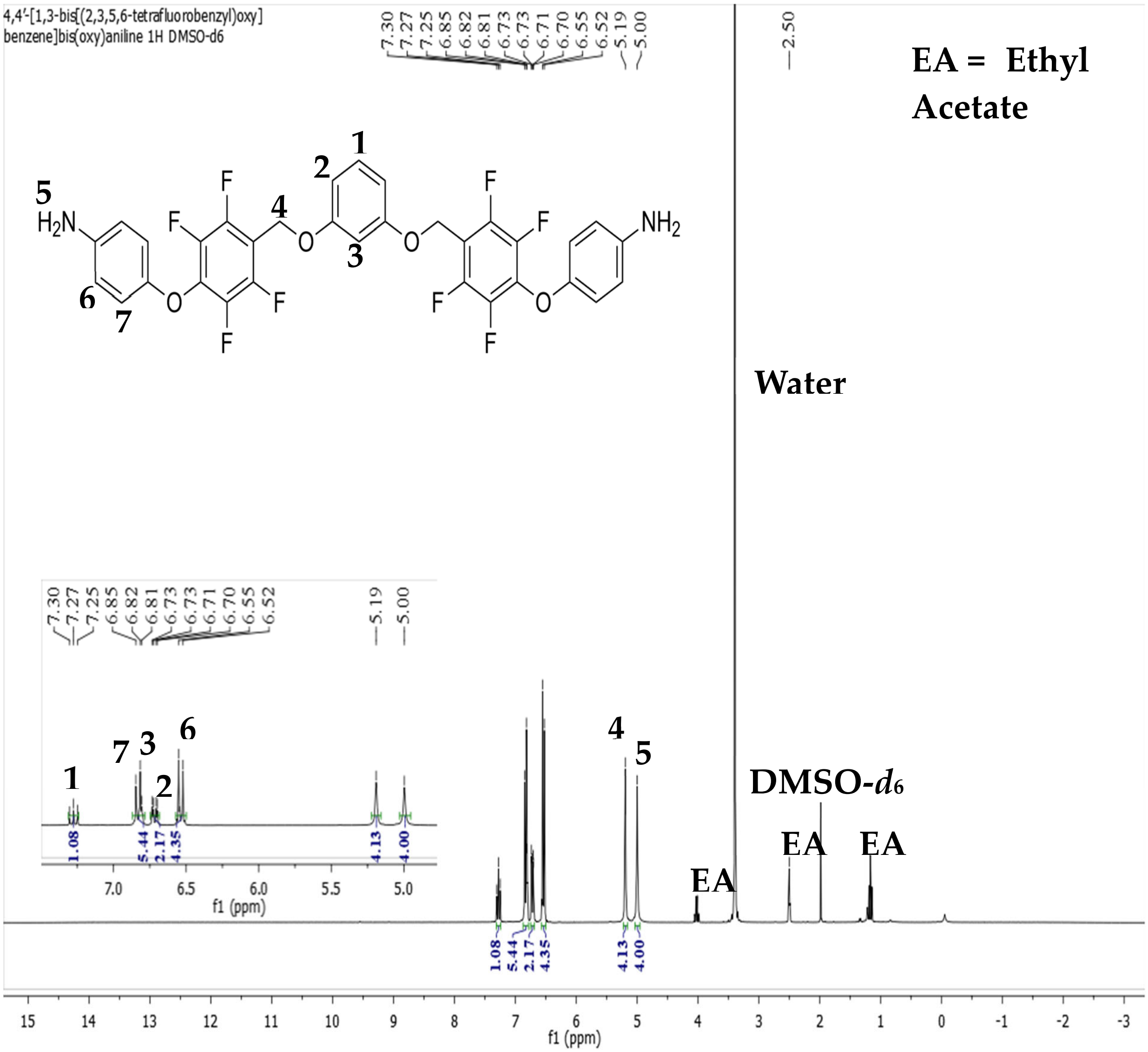

Figure S5. ¹H NMR Spectrum of 4,4′-[1,3-bis[(2,3,5,6-tetrafluorobenzyl)oxy]benzene]bis(oxy)aniline.

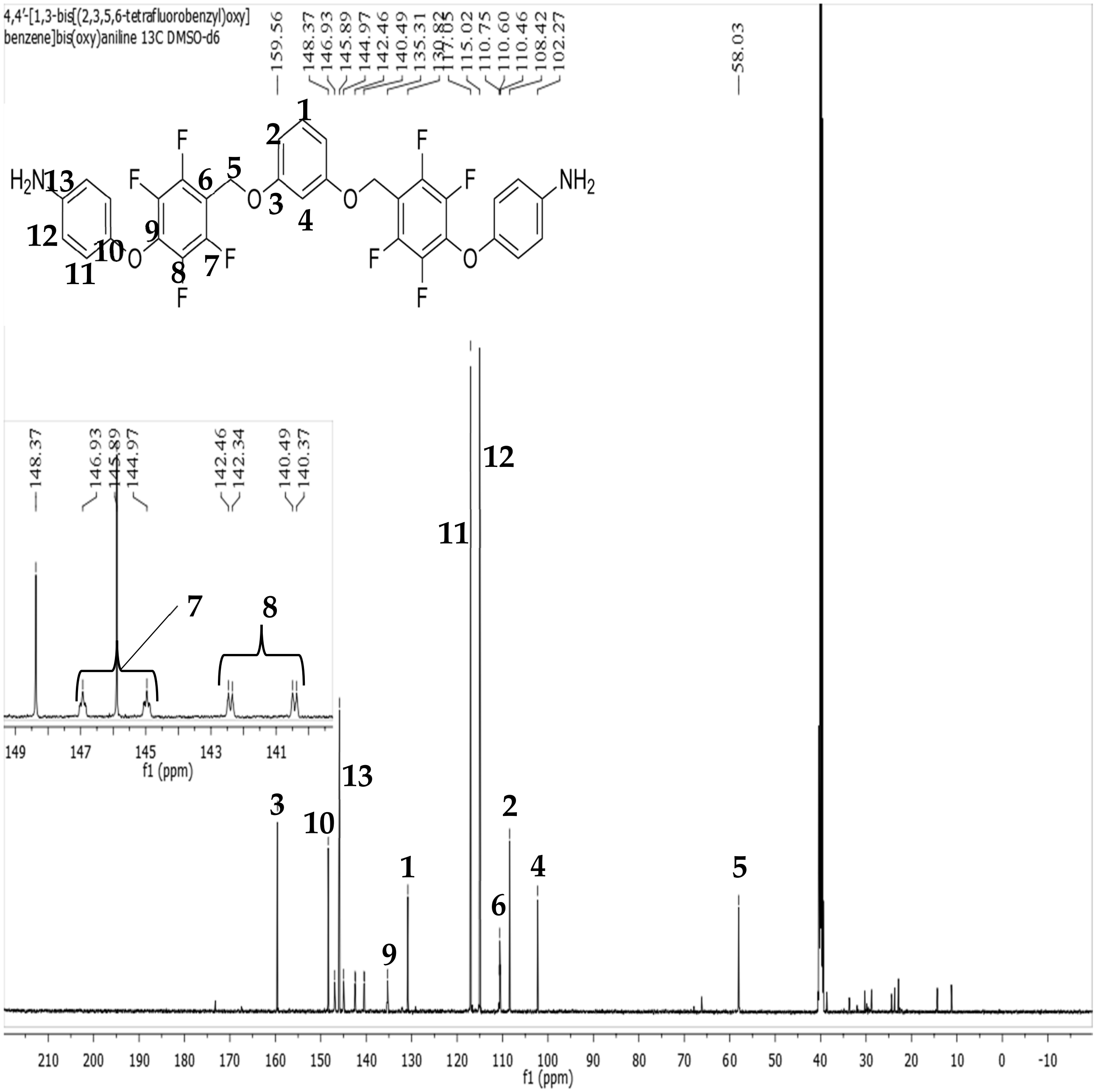

Figure S6. ^{13}C NMR spectrum of 4,4′-[1,3-bis[(2,3,5,6-tetrafluorobenzyl)oxy]benzene]bis(oxy)aniline.

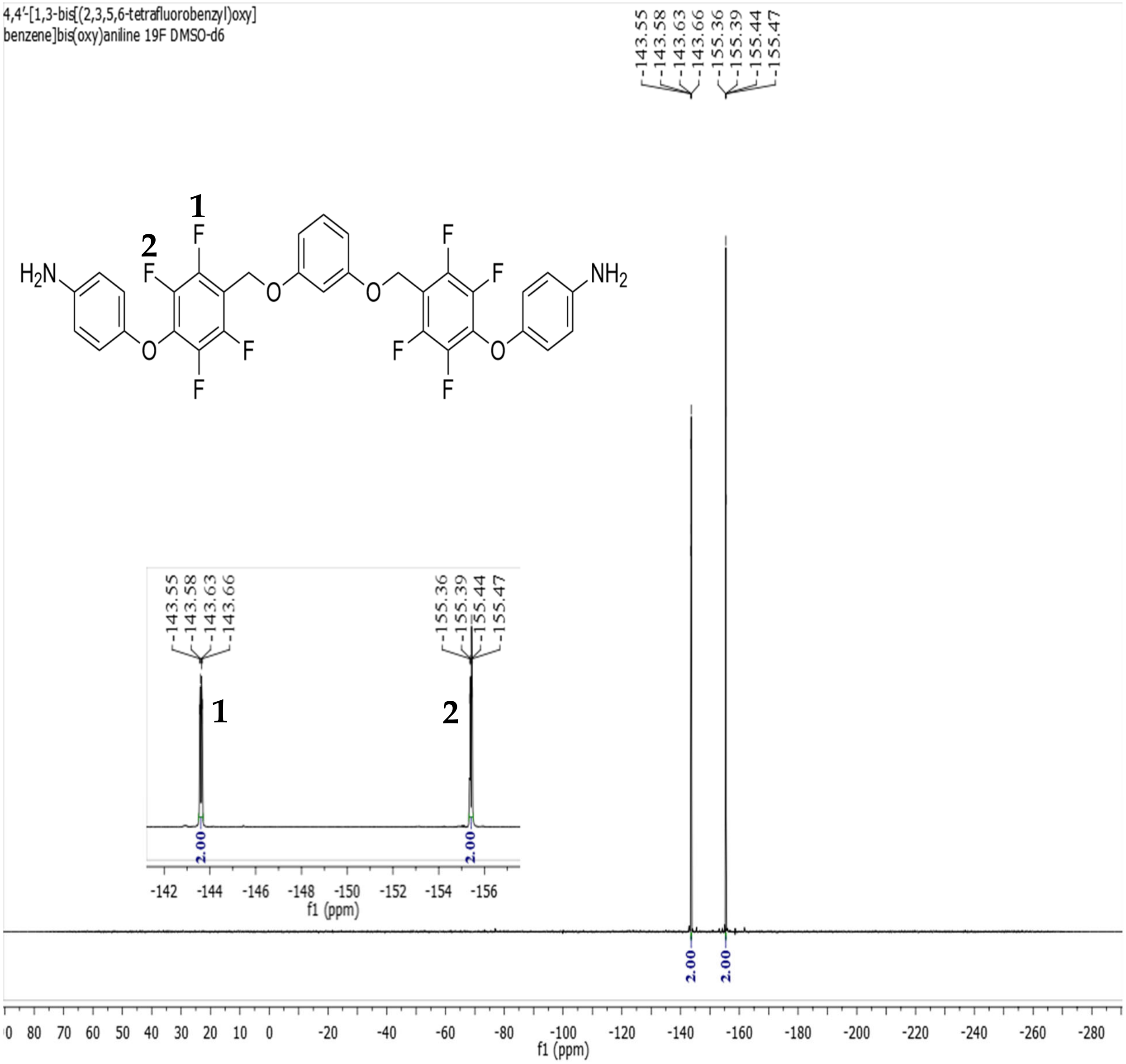

Figure S7. ^{19}F NMR Spectrum of 4,4′-[1,3-bis[(2,3,5,6-tetrafluorobenzyl)oxy]benzene]bis(oxy)aniline.

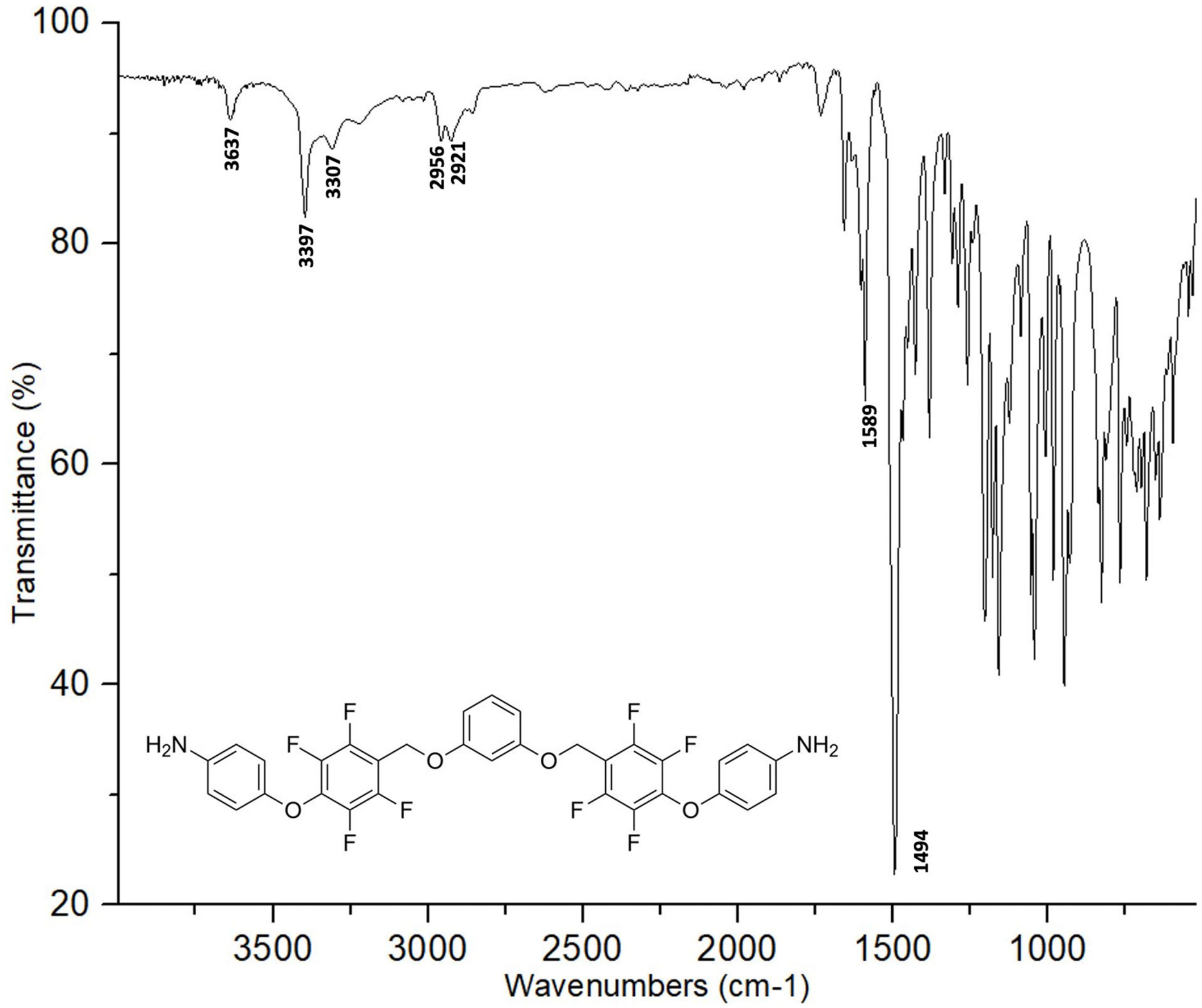

Figure S8. ATR FT-IR Spectrum of 4,4′-[1,3-bis[(2,3,5,6-tetrafluorobenzyl)oxy]benzene]bis(oxy)-aniline.

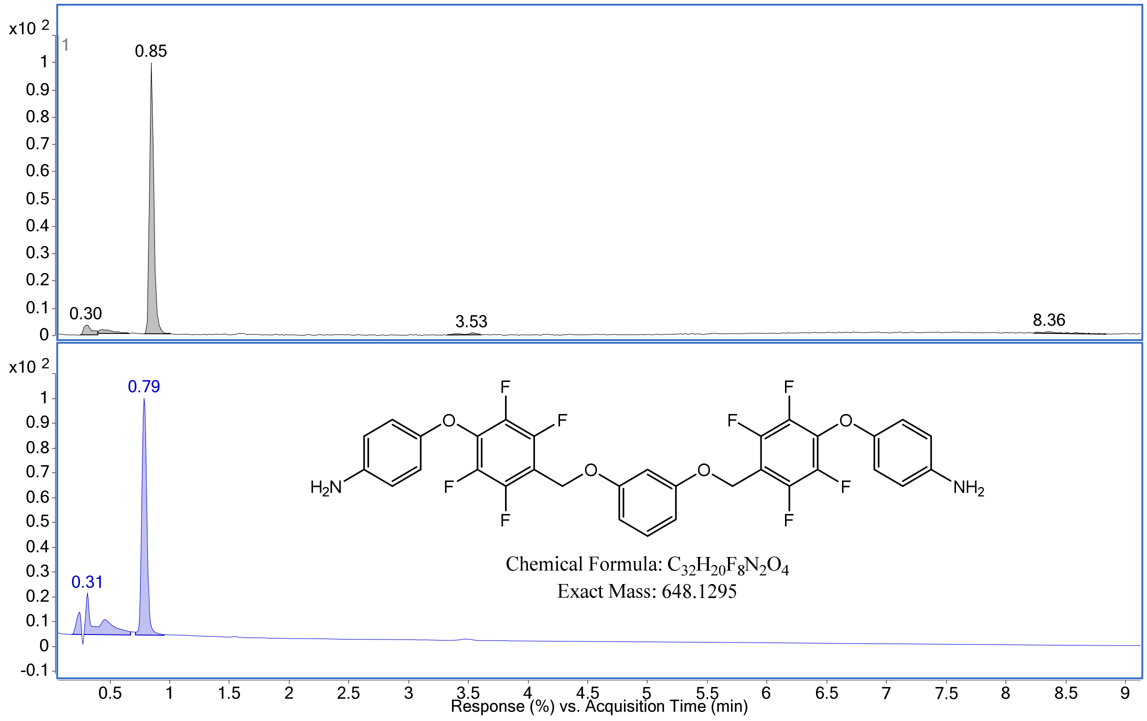

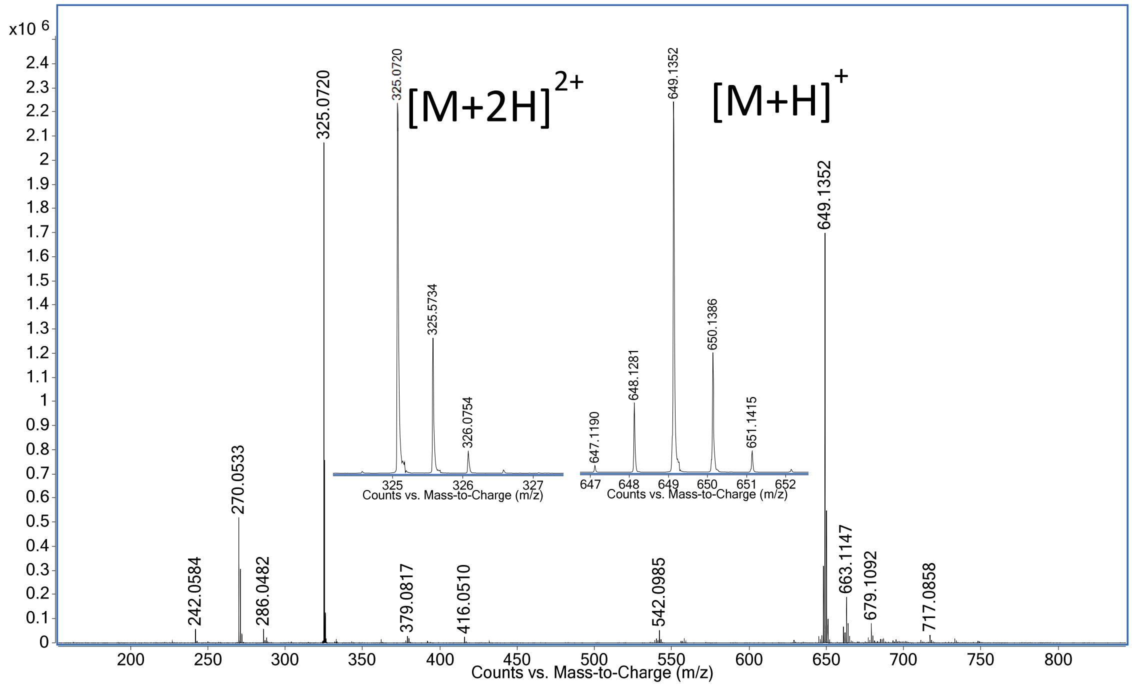

Figure S9. HRMS (LC-MS) of 4,4′-[1,3-bis[(2,3,5,6-tetrafluorobenzyl)oxy]benzene]bis(oxy)aniline. Top: ESI TIC and DAD chromatograms. Bottom: Mass spectra with enlargements of key ion clusters.

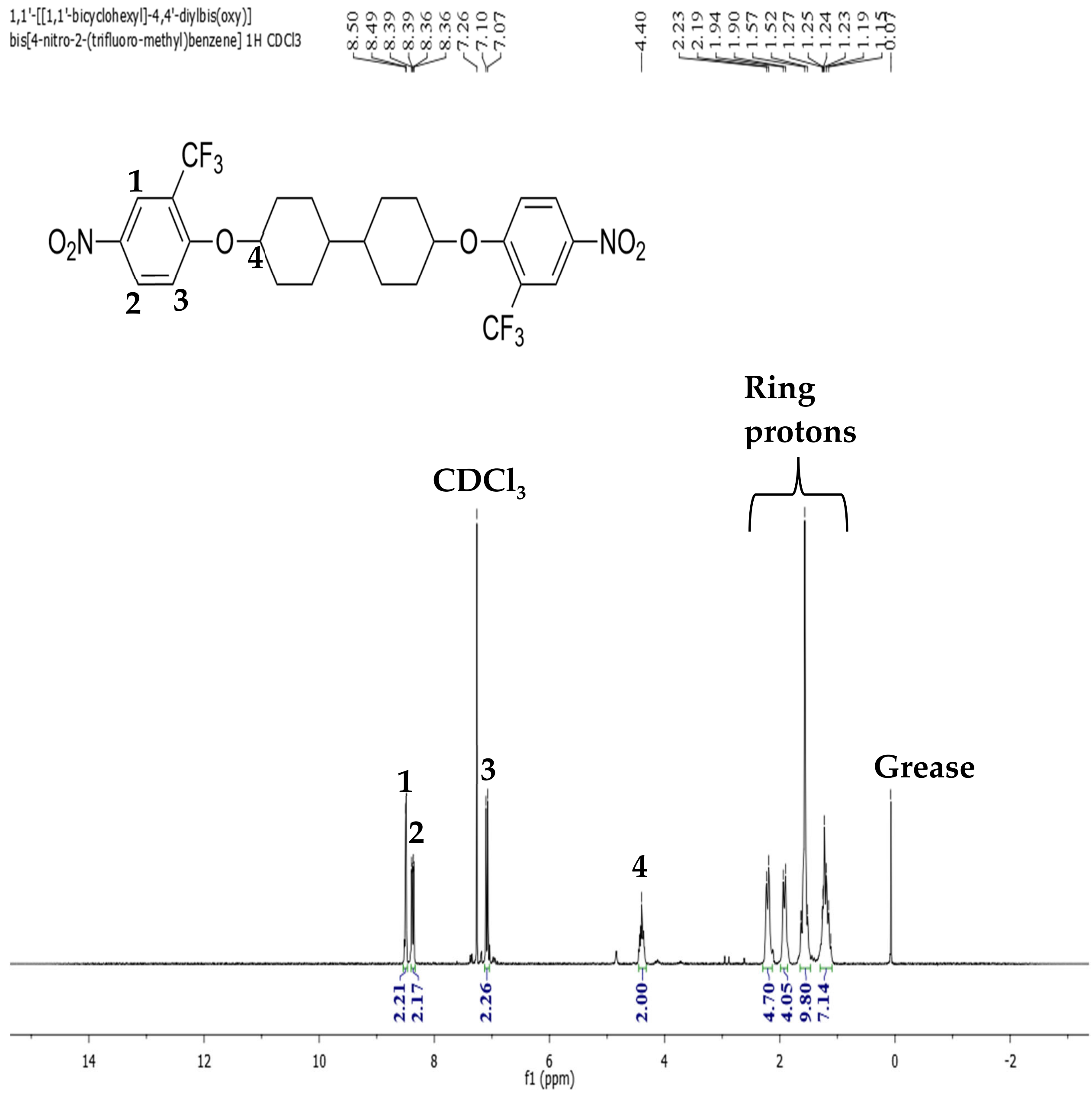

Figure S10. ^{1}H NMR Spectrum of 1,1'-[[1,1'-bicyclohexyl]-4,4'-diylbis(oxy)]bis[4-nitro-2-(trifluoro-methyl)benzene].

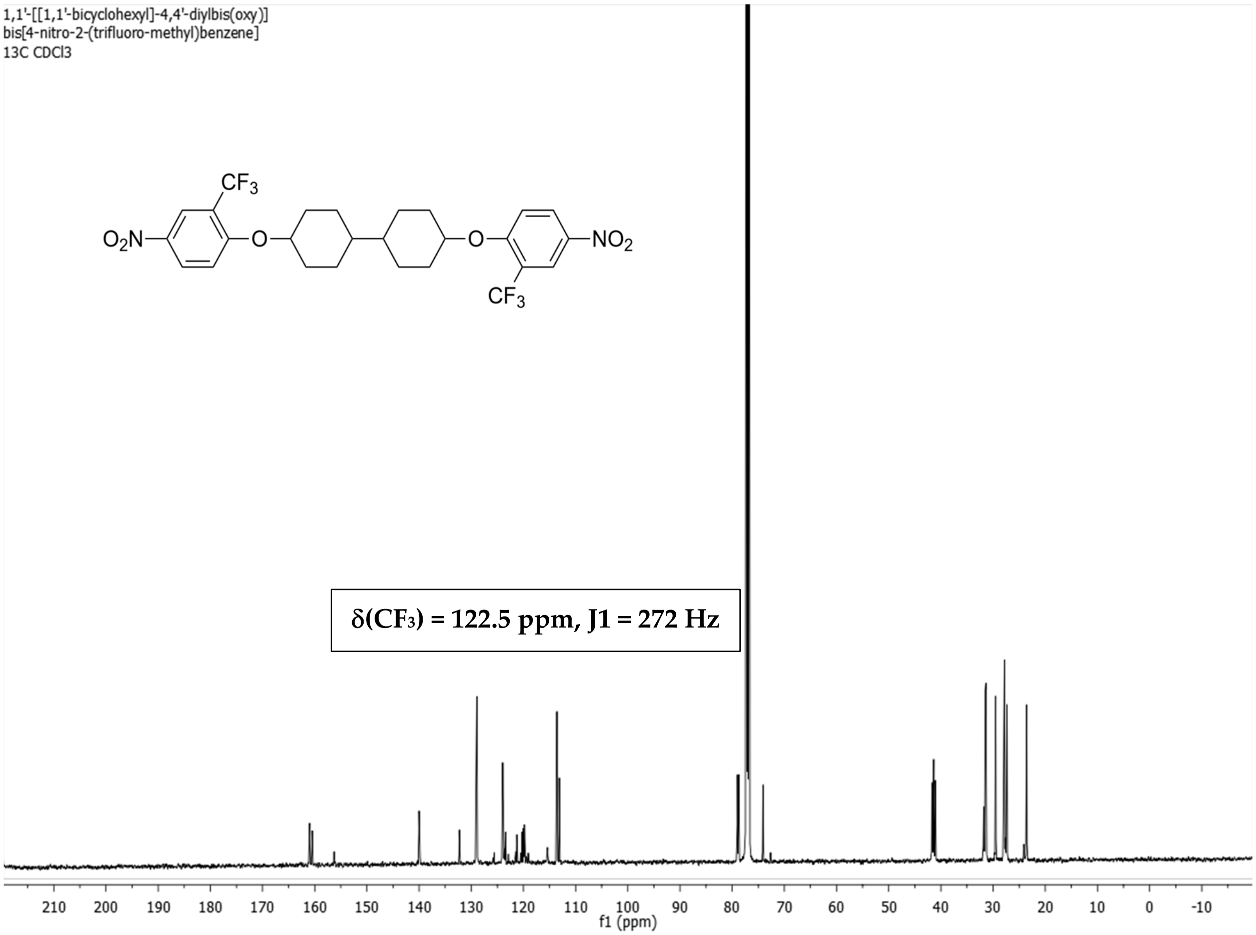

Figure S11. ^{13}C NMR spectrum of 1,1'-[[1,1'-bicyclohexyl]-4,4'-diylbis(oxy)]bis[4-nitro-2-(trifluoro-methyl)benzene].

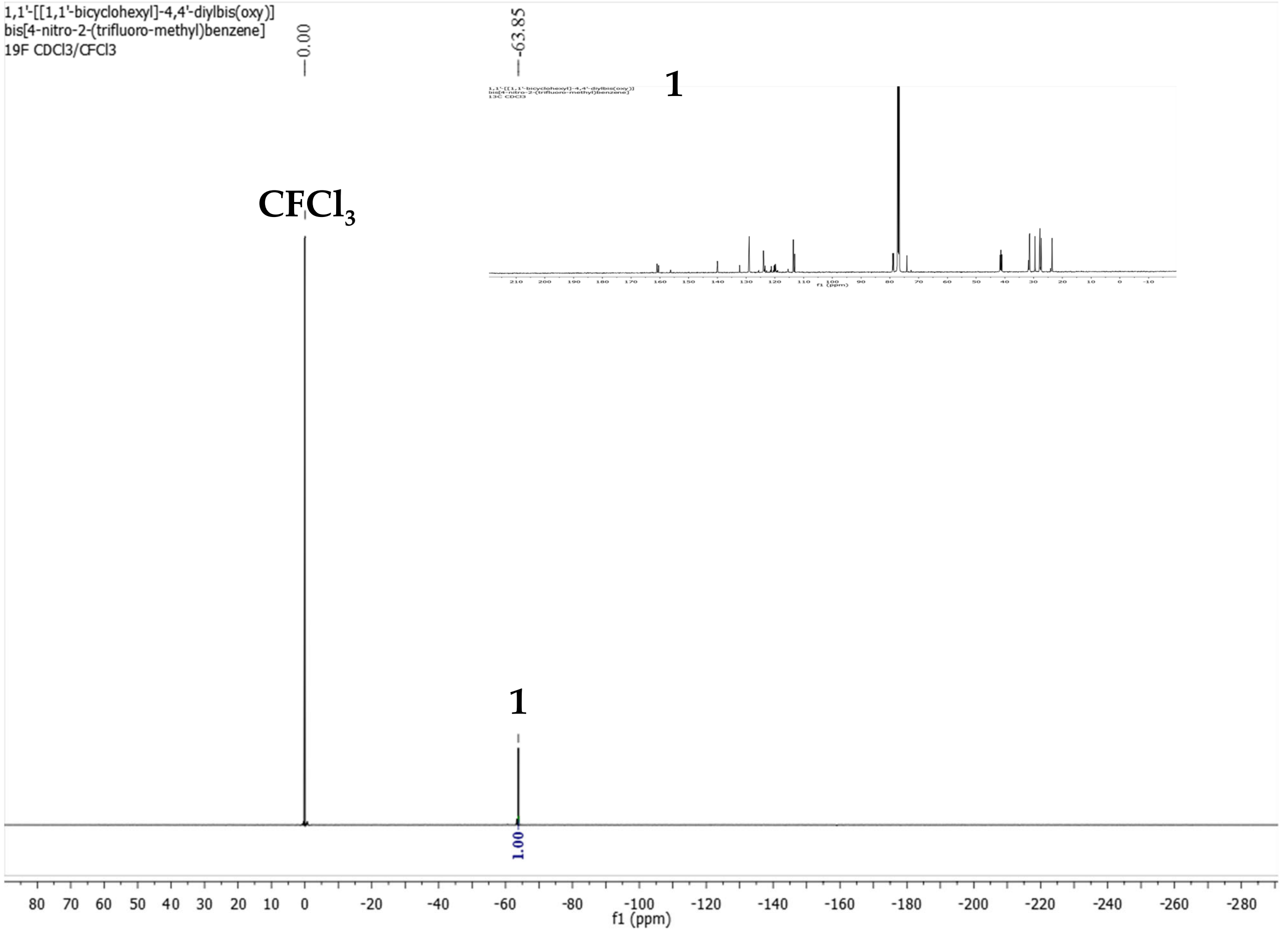

Figure S12. ^{19}F NMR Spectrum of 1,1'-[[1,1'-bicyclohexyl]-4,4'-diylbis(oxy)]bis[4-nitro-2-(trifluoro-methyl)benzene]..

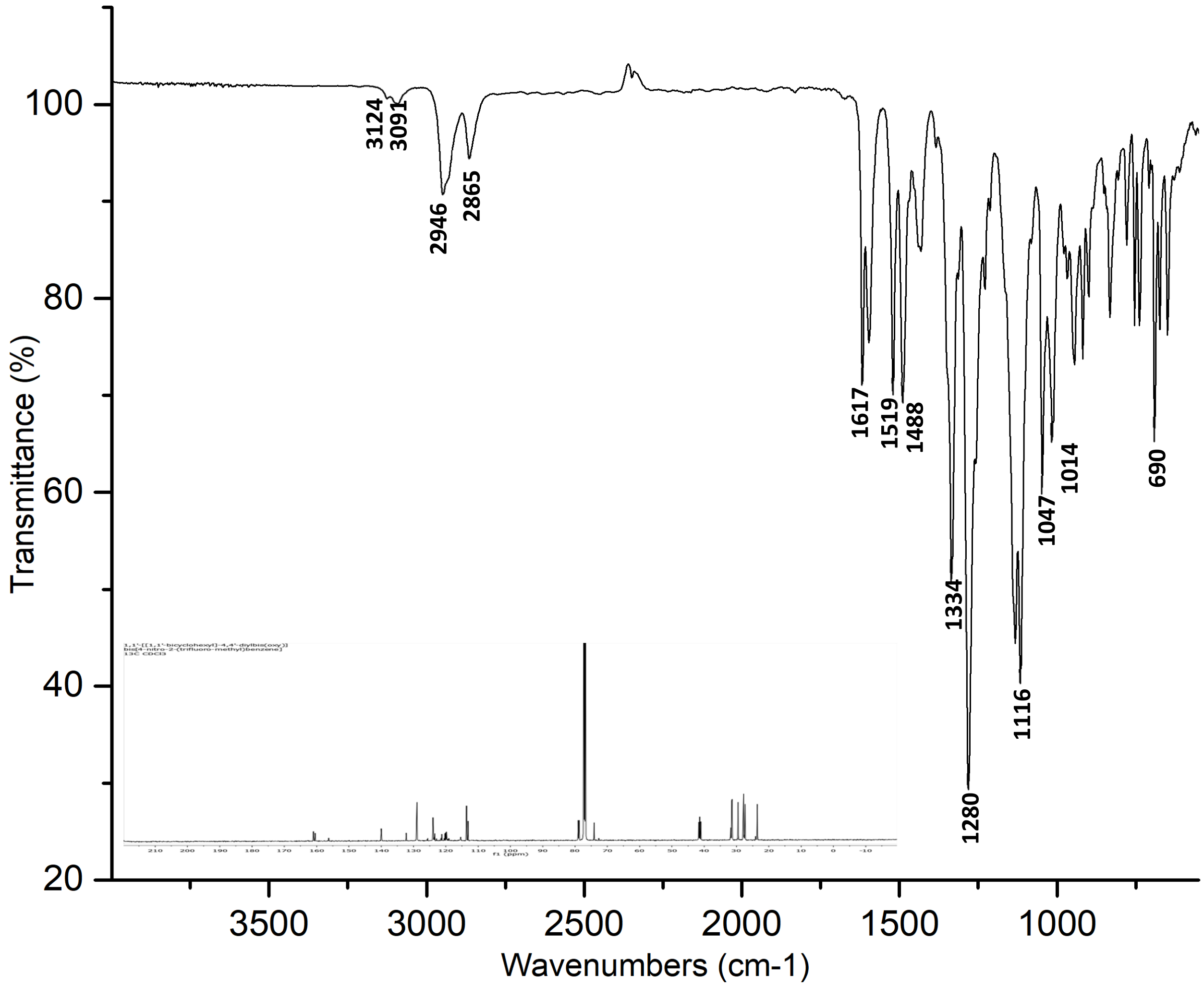

Figure S13. ATR FT-IR Spectrum of 1,1'-[[1,1'-bicyclohexyl]-4,4'-diylbis(oxy)]bis[4-nitro-2-(trifluoro-methyl)benzene].

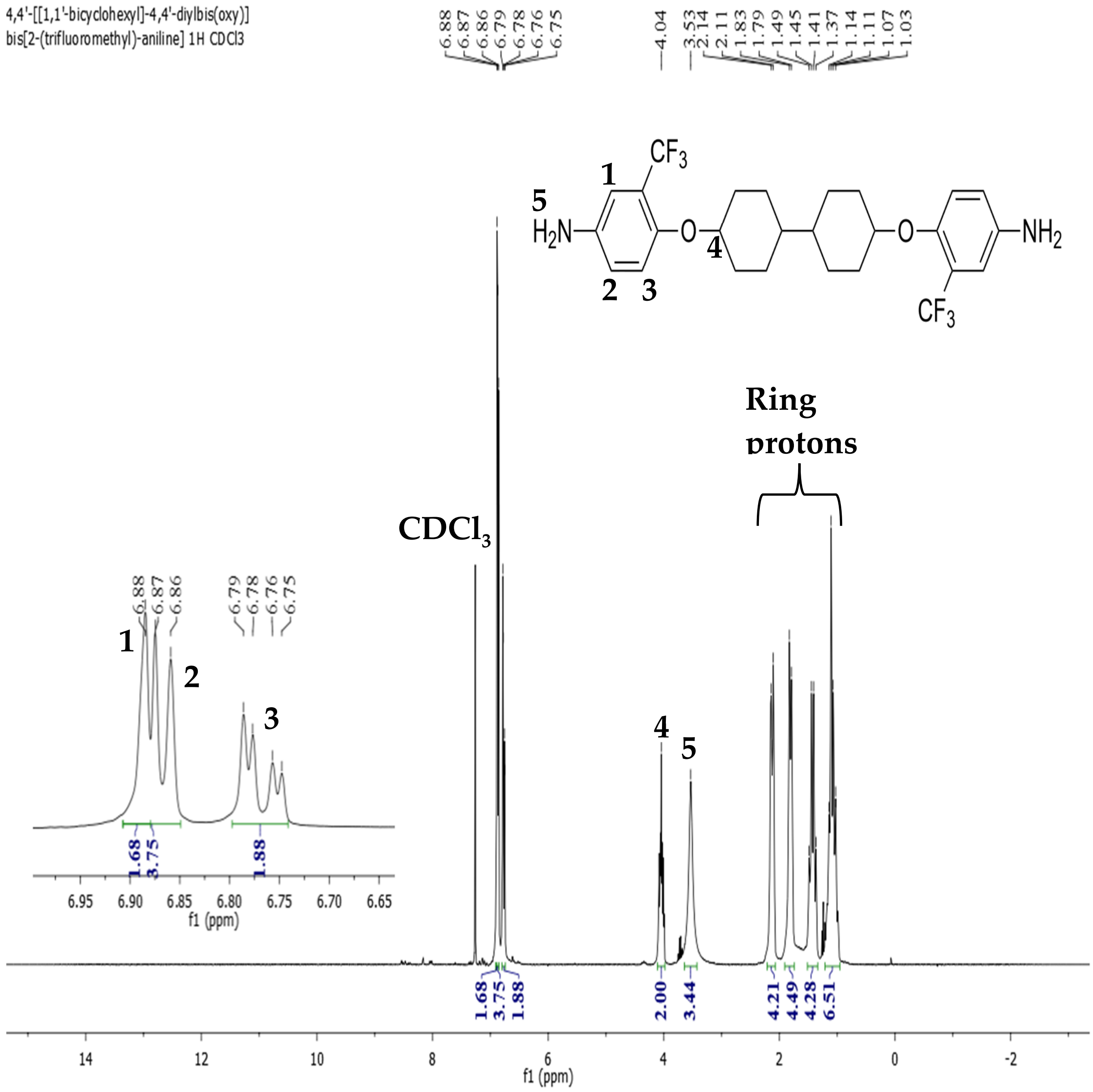

Figure S14. ^{1}H NMR Spectrum of 4,4'-[[1,1'-bicyclohexyl]-4,4'-diylbis(oxy)]bis[2-(trifluoromethyl)-aniline].

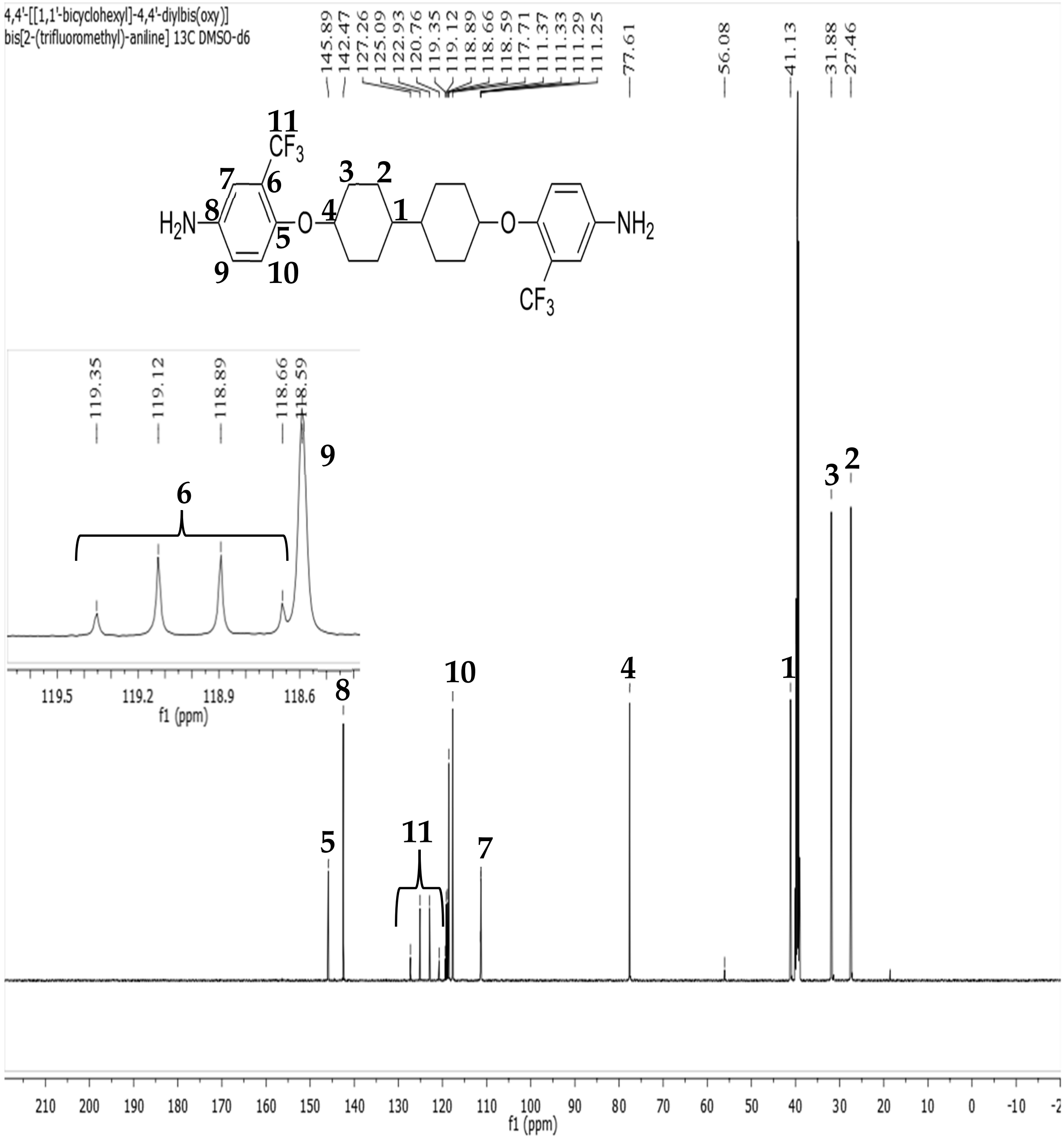

Figure S15. ^{13}C NMR spectrum of 4,4'-[[1,1'-bicyclohexyl]-4,4'-diylbis(oxy)]bis[2-(trifluoromethyl)-aniline].

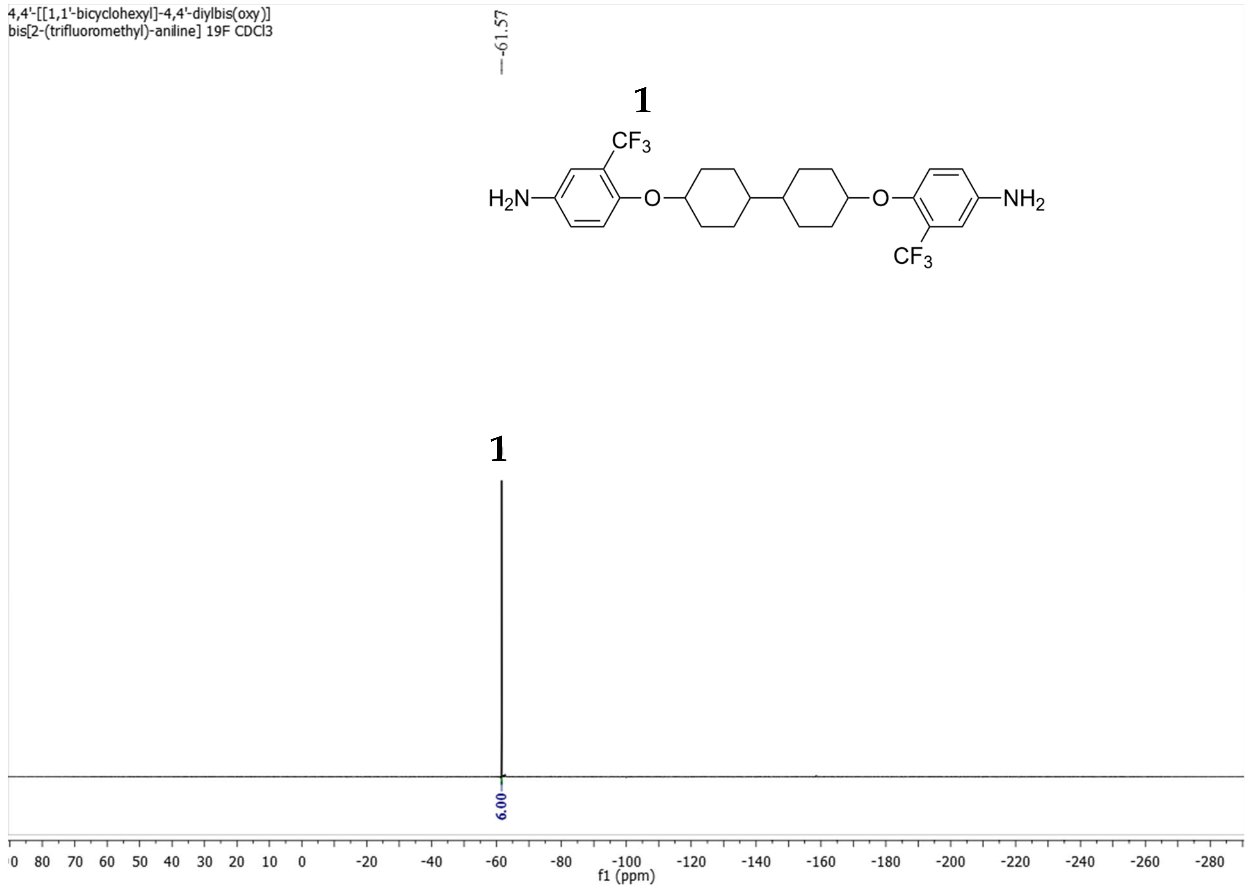

Figure S16. ^{19}F NMR Spectrum of 4,4'-[[1,1'-bicyclohexyl]-4,4'-diylbis(oxy)]bis[2-(trifluoromethyl)-aniline].

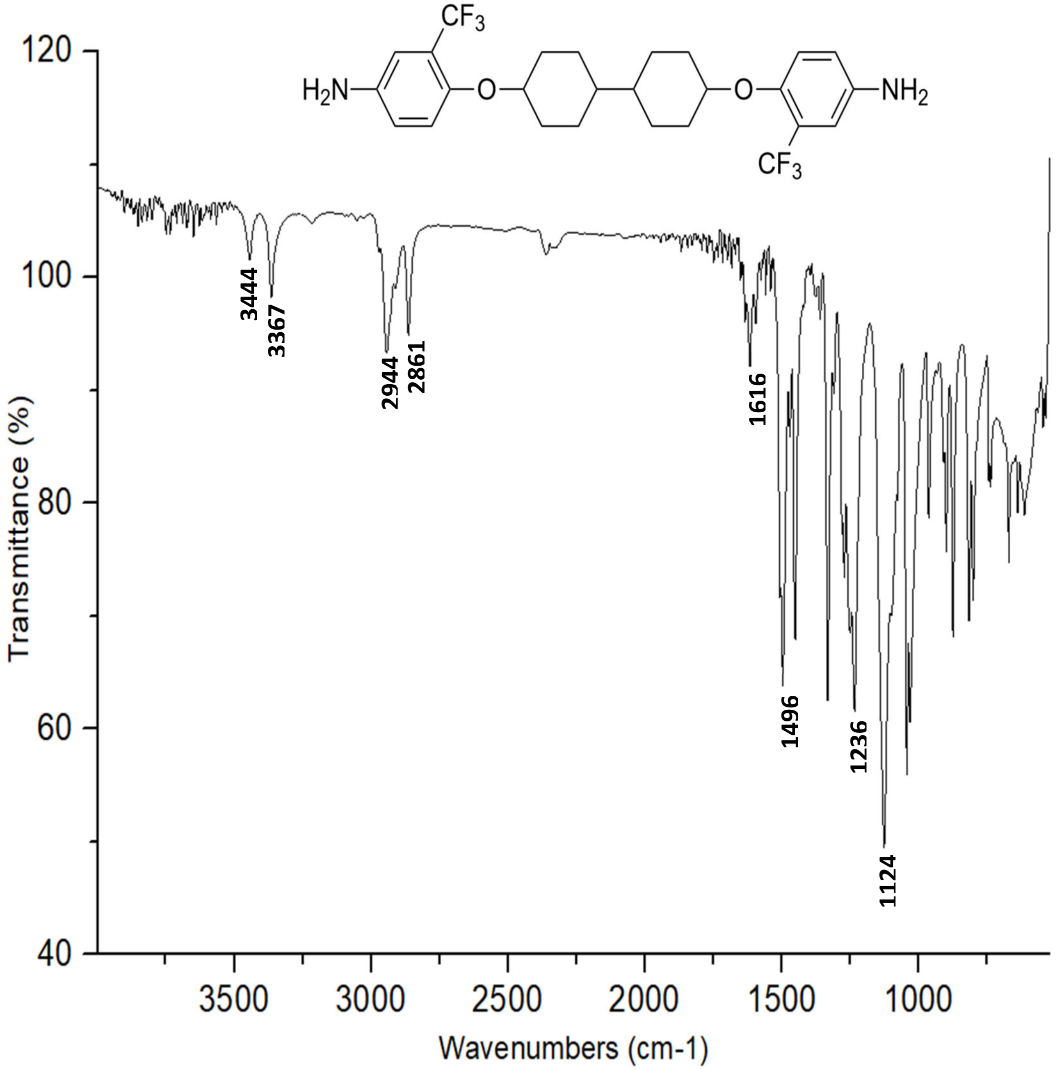

Figure S17. ATR FT-IR Spectrum of 4,4'-[[1,1'-bicyclohexyl]-4,4'-diylbis(oxy)]bis[2-(trifluoromethyl)-aniline].

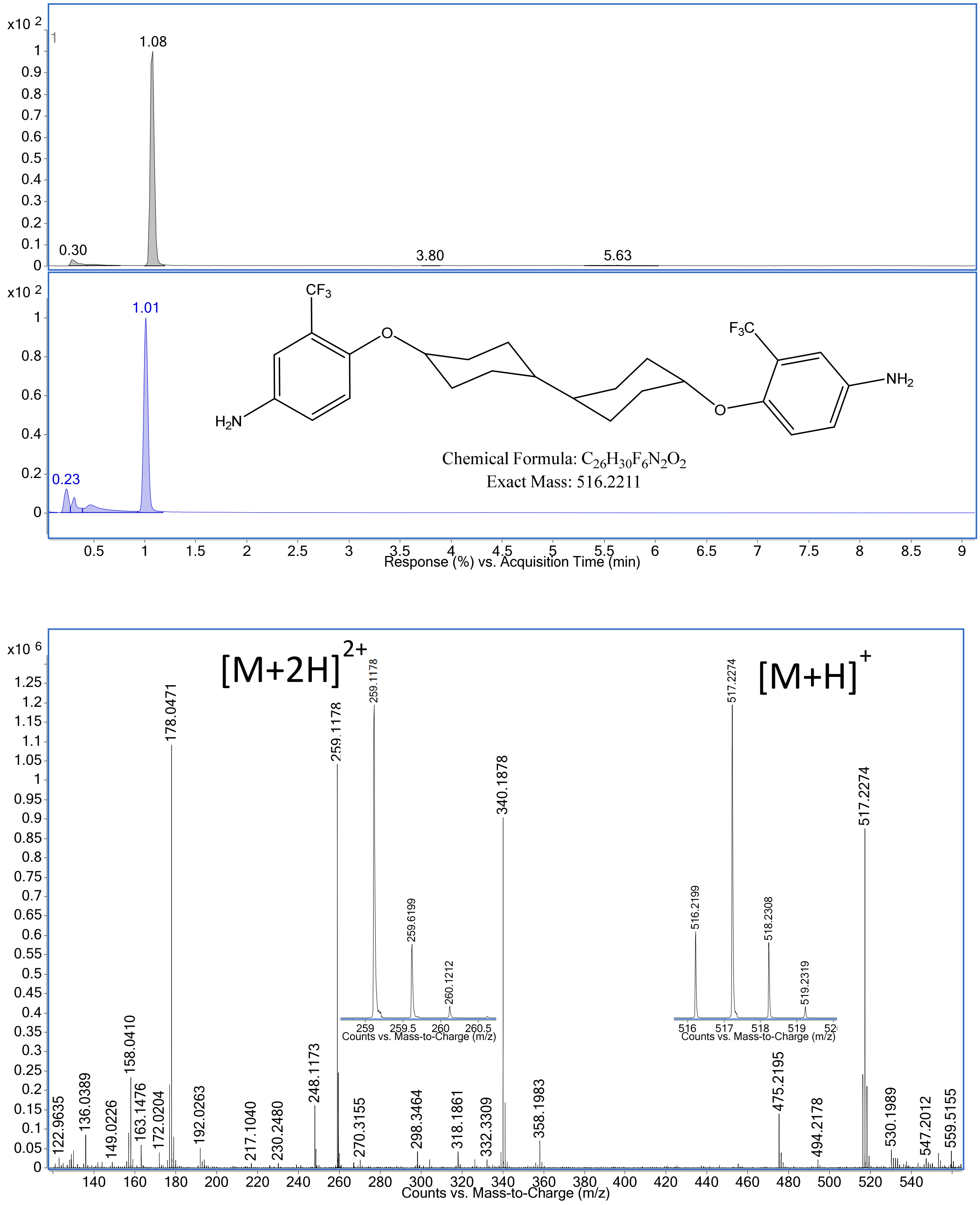

Figure S18. HRMS (LC-MS) of 4,4'-[[1,1'-bicyclohexyl]-4,4'-diylbis(oxy)]bis[2-(trifluoromethyl)-aniline]. Top: ESI TIC and DAD chromatograms. Bottom: Mass spectra with enlargements of key ion clusters.

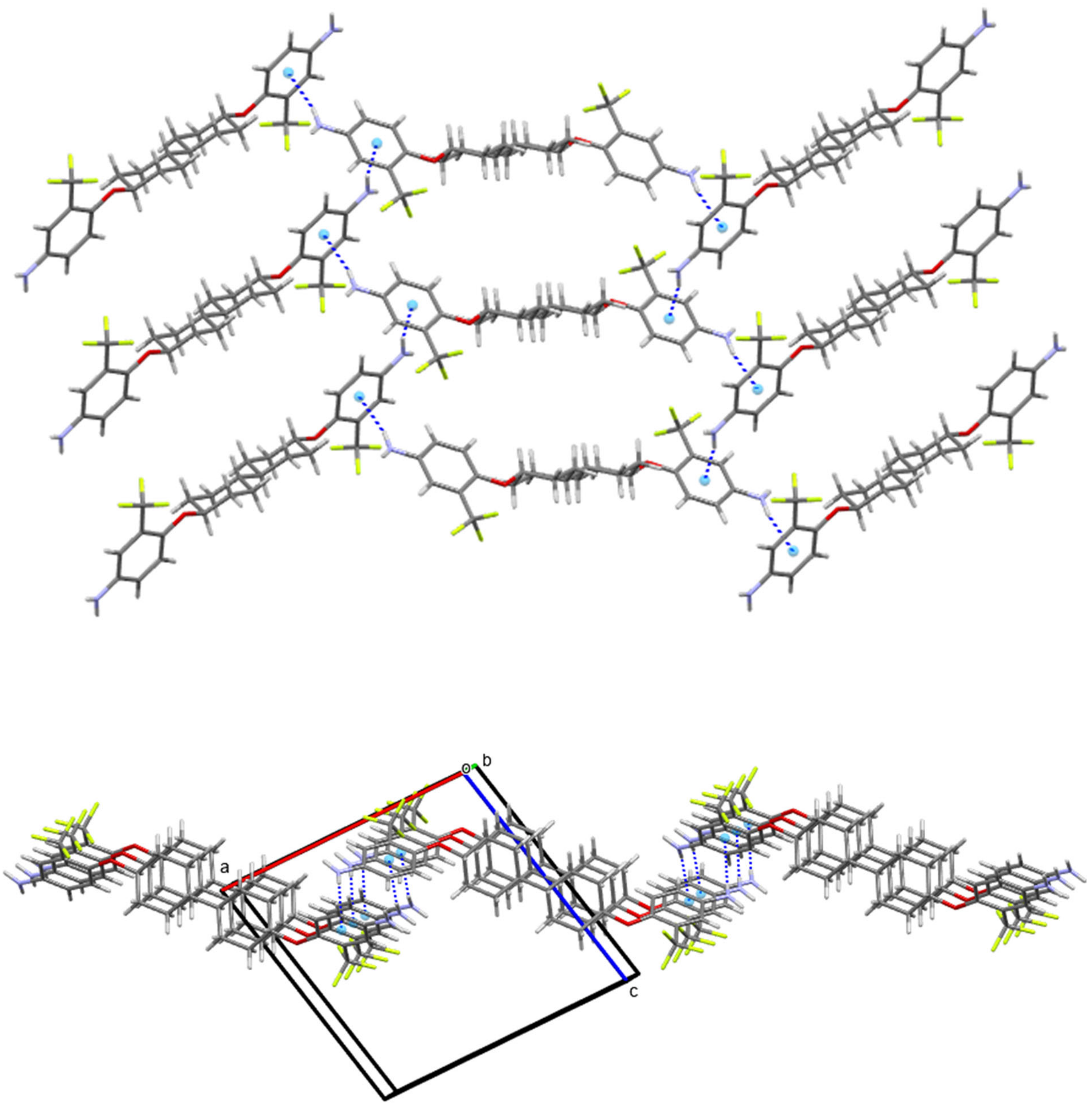

Figure S19. Hydrogen bonding in 4,4'-[[1,1'-bicyclohexyl]-4,4'-diylbis(oxy)]bis[2-(trifluoromethyl)aniline]. Formation of sheets parallel to (1 0 2) via intermolecular N-H···π interactions (blue dashed lines) in the structure of $C_{26}H_{30}F_6N_2O_2$. The centroids of the C7-C12 rings are shown as light blue spheres. Top: Viewed approximately normal to the plane of a sheet. Bottom: Viewed approximately along the plane of the sheet.

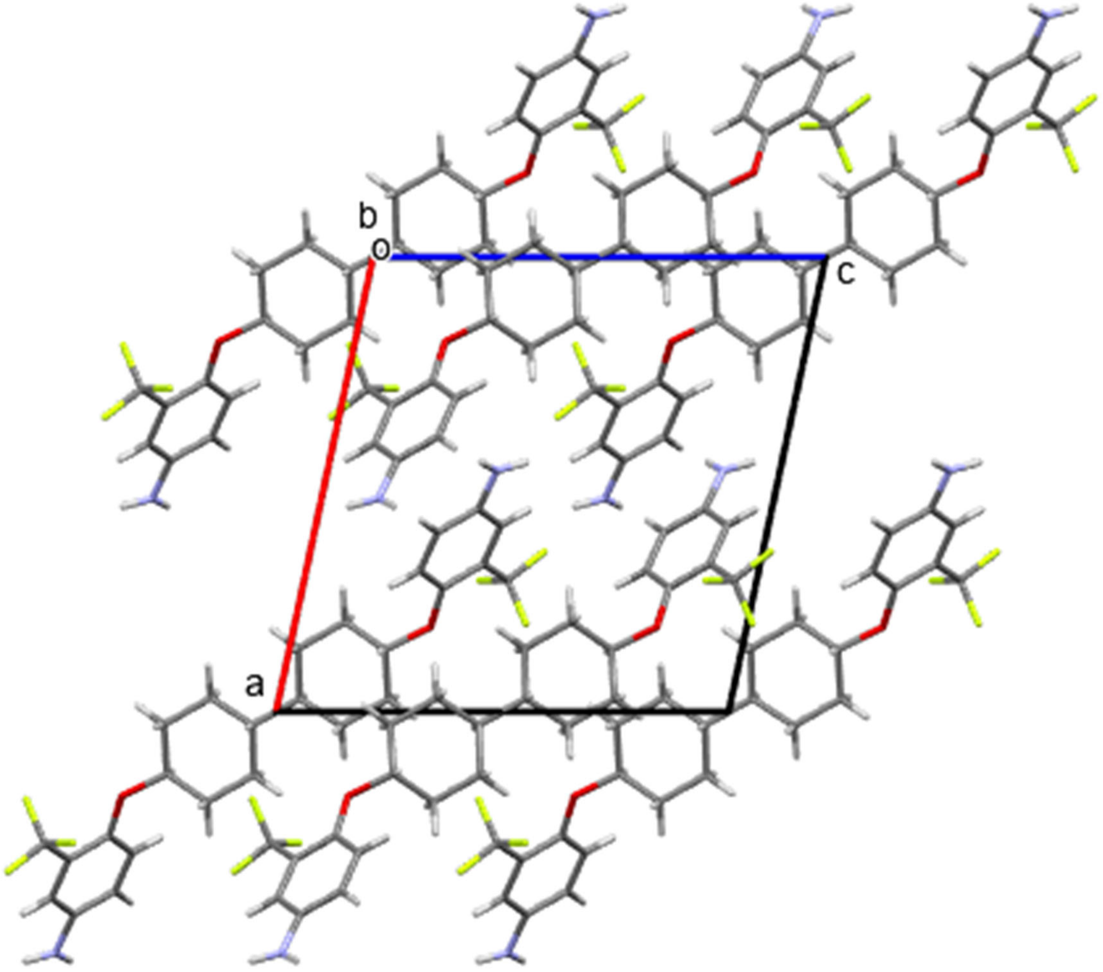

Figure S20. Unit cell packing diagram for 4,4'-[[1,1'-bicyclohexyl]-4,4'-diylbis(oxy)]bis[2-(trifluoromethyl)-aniline] ($C_{26}H_{30}F_6N_2O_2$, monomer 2) viewed along the *b*-axis. Carbon atoms are gray, nitrogen atoms are blue, oxygen atoms are red, fluorine atoms are green, and hydrogen atoms are white.

Table S1. Assignments of ^{13}C NMR spectrum of 1,3-bis[(pentafluorobenzyl)oxy]benzene from J-coupling to fluorine, NOEs, and additivity parameters [2].

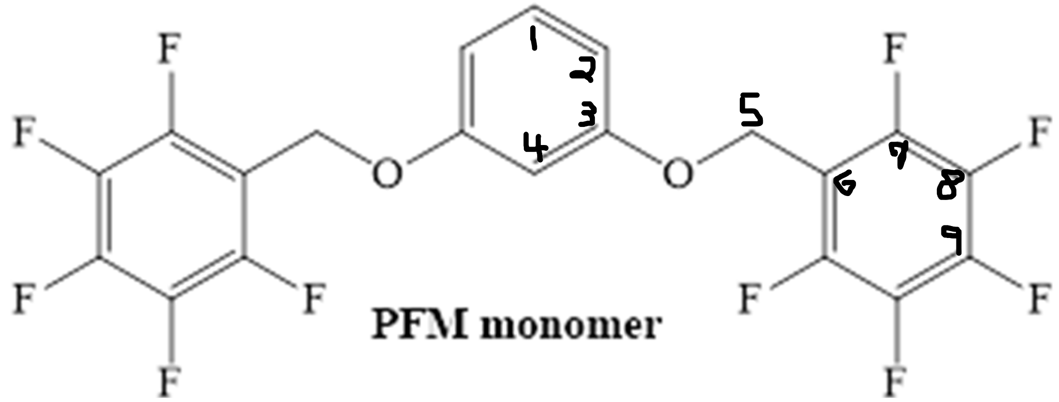

δ	Chemical Shift from Literature [1]	Chemical Shift from Experiment	Chemical Shift from Additivity Parameters	NOE or No NOE or J1 19F Coupling
**δ1**	130.6	130.73	130.5	NOE
**δ2**	108.3	108.40	106.4	NOE
**δ3**	159.4	159.40	160.9	No NOE
**δ4**	102.6	102.24	99.7	NOE
**δ5**	57.7	57.82	---	NOE
**δ6**	110.2	110.65	110.3	No NOE, J2 coupling
**δ7**	144.3, 147.6	145.59	149.5	No NOE, J1 19 F Coupling = 248.2 Hz
**δ8**	136.1, 139.5	137.51	134.4	No NOE, J1 19 F Coupling = 247.5 Hz
**δ9**	140.3, 143.7	141.49	140.5	No NOE, J1 19 F Coupling = 246.3 Hz

Table S2. Assignments of ^{13}C NMR spectrum of 4,4′-[1,3-bis[(2,3,5,6-tetrafluorobenzyl)-oxy]benzene]bis(oxy)aniline from J-coupling to fluorine, NOEs, and additivity parameters [2].

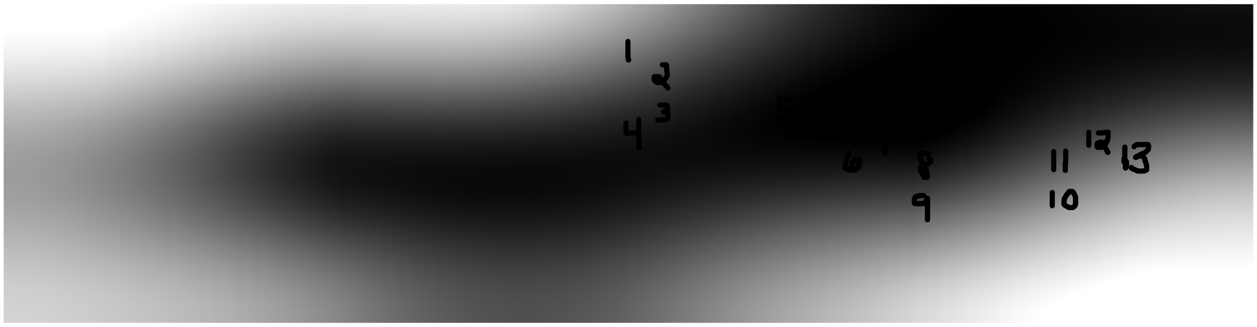

δ	Chemical Shift from Experiment (ppm)	Chemical Shift from Additivity Parameters (ppm)	NOE or No NOE or J1 (J2) 19F Coupling
**δ1**	130.82	130.5	NOE
**δ2**	108.42	106.4	NOE
**δ3**	159.56	160.9	No NOE
**δ4**	102.27	99.7	NOE
**δ5**	58.03	---	NOE
**δ6**	110.60	109.8	No NOE, J2 19F Coupling = 18.0 Hz
**δ7**	145.95	150.1	No NOE, J1 19 F Coupling = 245.0 Hz
**δ8**	141.42	138.3	No NOE, J1 19 F Coupling = 246.3 Hz
**δ9**	135.31	131.6	No NOE
**δ10**	148.37	147.7	No NOE
**δ11**	117.05	120.4	NOE
**δ12**	115.02	112.2	NOE
**δ13**	145.89	141.5	No NOE

Table S3. Assignments of ^{13}C NMR spectrum of 4,4'-[[1,1'-bicyclohexyl]-4,4'-diylbis(oxy)]bis[2-(trifluoromethyl)aniline] from J-coupling to fluorine, NOEs, and additivity parameters [2].

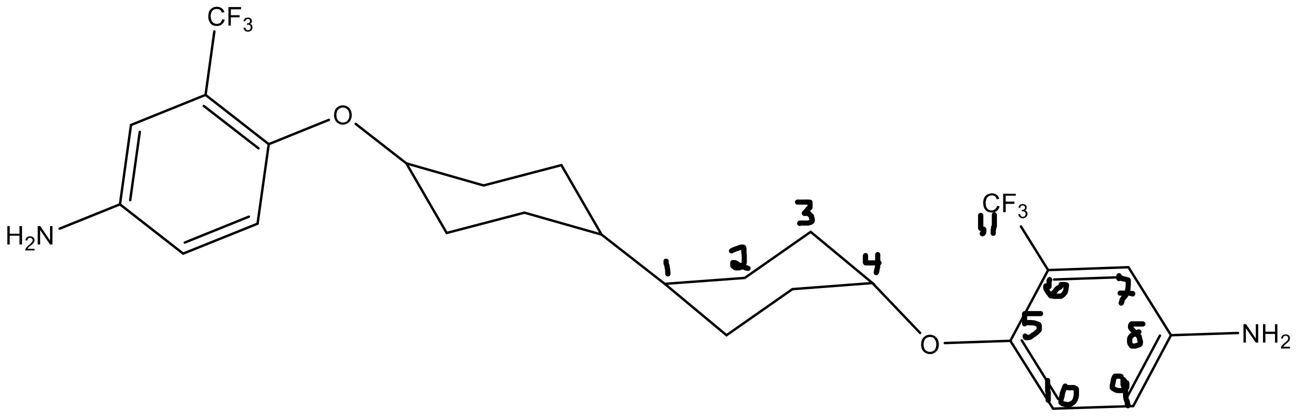

Chemical Formula: $C_{26}H_{30}F_6N_2O_2$
Exact Mass: 516.22

δ	Chemical Shift from Experiment	Chemical Shift from Additivity Parameters	NOE or No NOE or J1 (J2) 19F Coupling
**δ1**	41.13	31.3	NOE
**δ2**	27.46 or 31.88	33.3	NOE
**δ3**	31.88 or 27.46	31.3	NOE
**δ4**	77.61	79.1	NOE
**δ5**	145.89	147.9	No NOE
**δ6**	119.01 ($^2J_{C-F}$)	106.0	No NOE, J2 19F Coupling = 28.8 Hz
**δ7**	111.31	114.0	NOE, , J3 19F Coupling = 2.5 Hz
**δ8**	142.47	139.1	No NOE
**δ9**	118.59	119.4	NOE
**δ10**	117.71	115.3	NOE
**δ11**	124.01 ($^1J_{C-F}$)	Around 110-120 ppm, large quartet due to coupling	No NOE, J1 19F Coupling = 272.5 Hz

4. References

1. Quast, M. J.; Mueller, A. Hyperbranched Polyfluorinated Benzyl Ether Polymers : Mechanism, Kinetics , and Optimization. *J. Polym. Sci., Part A: Polym. Chem.* **2014**, *52*, 985–994.
2. Wehrli, F. W.; Wirthlin, T. *Interpretation of Carbon-13 NMR Spectra*. Heyden & Sons Inc.: Philadelphia, 1980; pp 43-47.
